# Chamber-enriched gene expression profiles in failing human hearts with reduced ejection fraction

**DOI:** 10.1038/s41598-021-91214-2

**Published:** 2021-06-04

**Authors:** Xin Luo, Jun Yin, Denise Dwyer, Tracy Yamawaki, Hong Zhou, Hongfei Ge, Chun-Ya Han, Artem Shkumatov, Karen Snyder, Brandon Ason, Chi-Ming Li, Oliver Homann, Marina Stolina

**Affiliations:** 1 Genome Analysis Unit, Amgen Research, 1120 Veterans BLVD, South San Francisco, CA 94010 USA; 2 Department of Cardiometabolic Disorders, Amgen Research, One Amgen Center Drive, Thousand Oaks, CA 91320 USA; 3 Department of Cardiometabolic Disorders, Amgen Research, 1120 Veterans BLVD, South San Francisco, CA 94010 USA; 4 TS&BA Pathology, Amgen Research, 1120 Veterans BLVD, South San Francisco, CA 94010 USA; 5 Clinical Biomarkers, Amgen Research, 1120 Veterans BLVD, South San Francisco, CA 94010 USA

**Keywords:** Heart failure, Gene expression, Gene regulatory networks

## Abstract

Heart failure with reduced ejection fraction (HFrEF) constitutes 50% of HF hospitalizations and is characterized by high rates of mortality. To explore the underlying mechanisms of HFrEF etiology and progression, we studied the molecular and cellular differences in four chambers of non-failing (NF, n = 10) and HFrEF (n = 12) human hearts. We identified 333 genes enriched within NF heart subregions and often associated with cardiovascular disease GWAS variants. Expression analysis of HFrEF tissues revealed extensive disease-associated transcriptional and signaling alterations in left atrium (LA) and left ventricle (LV). Common left heart HFrEF pathologies included mitochondrial dysfunction, cardiac hypertrophy and fibrosis. Oxidative stress and cardiac necrosis pathways were prominent within LV, whereas TGF-beta signaling was evident within LA. Cell type composition was estimated by deconvolution and revealed that HFrEF samples had smaller percentage of cardiomyocytes within the left heart, higher representation of fibroblasts within LA and perivascular cells within the left heart relative to NF samples. We identified essential modules associated with HFrEF pathology and linked transcriptome discoveries with human genetics findings. This study contributes to a growing body of knowledge describing chamber-specific transcriptomics and revealed genes and pathways that are associated with heart failure pathophysiology, which may aid in therapeutic target discovery.

## Introduction

By physiological function, the human heart is a muscular pump that circulates blood, perfusing tissues throughout the body. The four heart chambers have complex tissue structure and cell composition: atria and ventricles are thin- and thick-walled chambers, respectively. These morphological differences reflect differences in the amount of myocardium and the force required for each chamber to perform its function^1^. The unique cellular composition of each chamber is determined during embryogenesis by three progenitor cell populations—cardiac mesenchymal progenitors (contributing to cardiomyocytes, conduction system cells and endocardial cell development), cardiac and neural crest cells (contributing to smooth muscle cells and parasympathetic neural cells) and pro-epicardium (contributing to cardiomyocytes, endothelial cells, coronary smooth muscle cells and cardiac fibroblasts)^2^. Given that the diverse spectrum of disorders affecting the heart can only occasionally be traced to a specific single genetic mutation, it is important to determine changes within cardiac tissue at the molecular, cellular and genetic level in order to identify both genes and pathways that could influence heart failure progression. The most abundant cells within adult human heart are cardiomyocytes, fibroblasts, endothelial cells, and perivascular cells^3^. All major cardiac tissue cell types respond to physiological and pathological stress and participate in the pathogenesis of heart failure^3^.

Heart failure (HF) is a common clinical disorder that impairs the ability of the ventricle to fill with or eject blood and is associated with significant morbidity and mortality for both women and men^4^, and accounts for approximately 8% of all cardiovascular deaths^5^. HF due to left ventricular (LV) dysfunction is categorized according to LV ejection fraction (LVEF). HF with reduced ejection fraction (HFrEF) represents approximately 50% of all HF cases, affects more men than women, and has significant morbidity and mortality for all patients^6^.

**Figure 1:**
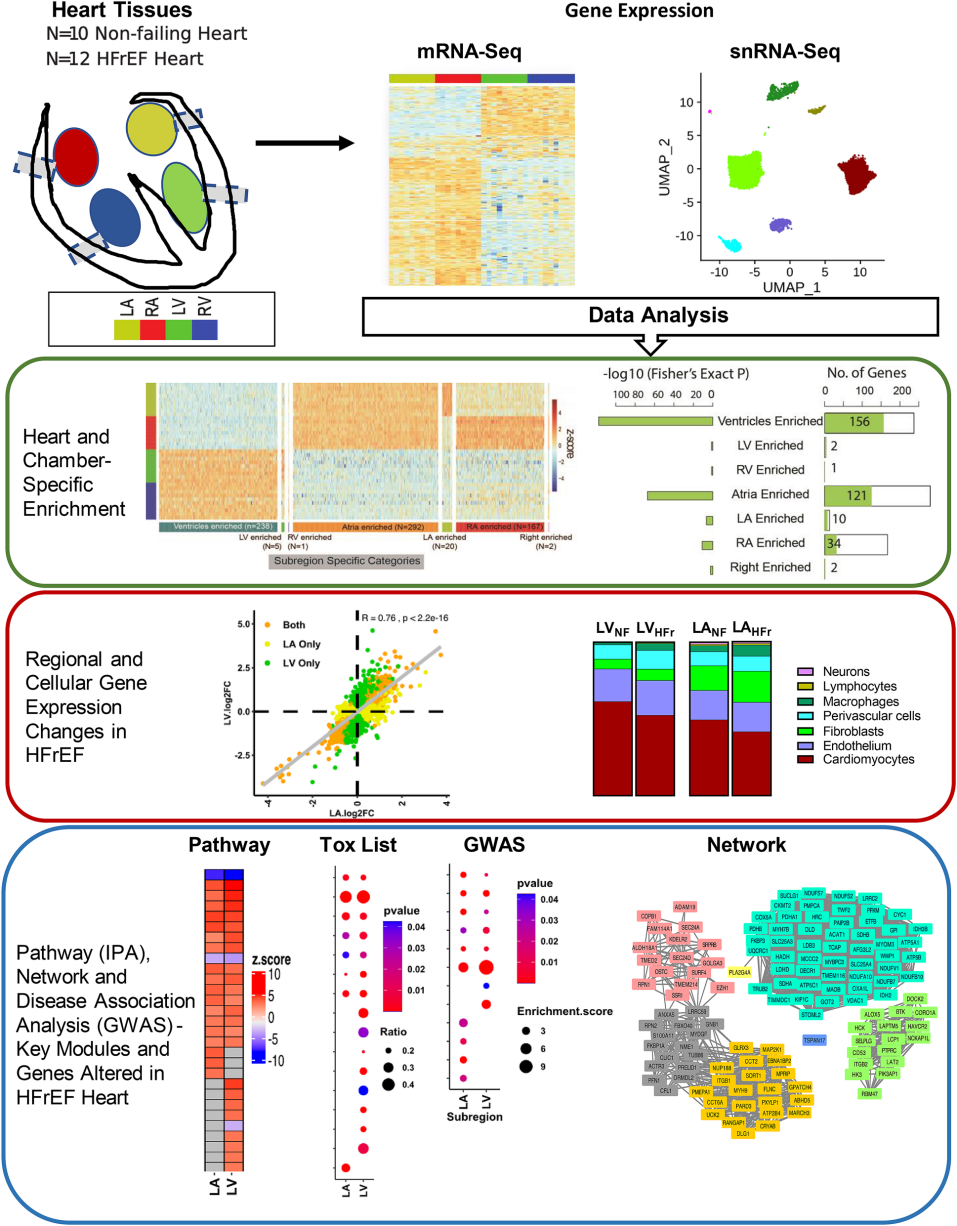
Graphic abstract of study design and flow chart.

Expression profiling of non-failing hearts^7–13^ and HFrEF-diseased hearts^12–18^ by transcriptomics and proteomics has enhanced our understanding of normal cardiac function, development, and shed light on HFrEF-related changes. Most human HFrEF RNA-Seq studies were focused on regional changes within the LV. HFrEF-related gene expression changes in other heart chambers have not been thoroughly explored, despite the known clinical link between the left atrium (LA) and HFrEF prognosis^19,20^. Recent advances and application of single cell technology in cardiac tissues have revealed cellular expression network heterogeneity and led to novel insights of cardiac physiology and pathology^11–13,21^. Thus, to better understand the underlying mechanisms of HFrEF etiology and progression, we performed a systematic analysis combining the precision of single cell technology together with global transcriptome profiling of all four heart chambers from 10 non-failing (NF) and 12 HFrEF donors (Fig. 1). Furthermore, we characterized the heterogenous nature of HFrEF among patients and across cell types and explored the underlying gene regulatory network, signaling pathways and genetic associations (Fig. 1).

## Results

### Subregion-Enriched and Heart-Enriched genes in human heart tissue

Ten non-failing (NF) adult heart samples were obtained from six male and four female deceased donors with mean age of 45, mean left ventricle ejection fraction (LVEF) of 61.3%, and mean BMI of 33.1. There was no indication of heart failure or related pathology within the donor’s medical record (Supplemental Table 1). Twelve adult heart samples were obtained from seven male and five female deceased HFrEF donors with mean age of 39, mean LVEF of 34.9%, and mean BMI of 25.9 (Supplemental Table 1).

All samples were extracted from each of the four heart chambers: left ventricle (LV), right ventricle (RV), left atrium (LA) and right atrium (RA). RNA-Seq profiling allowed us to characterize the expression landscape of each NF and HFrEF chamber and to define regional changes in gene expression associated with impaired cardiac function.

**Figure 2:**
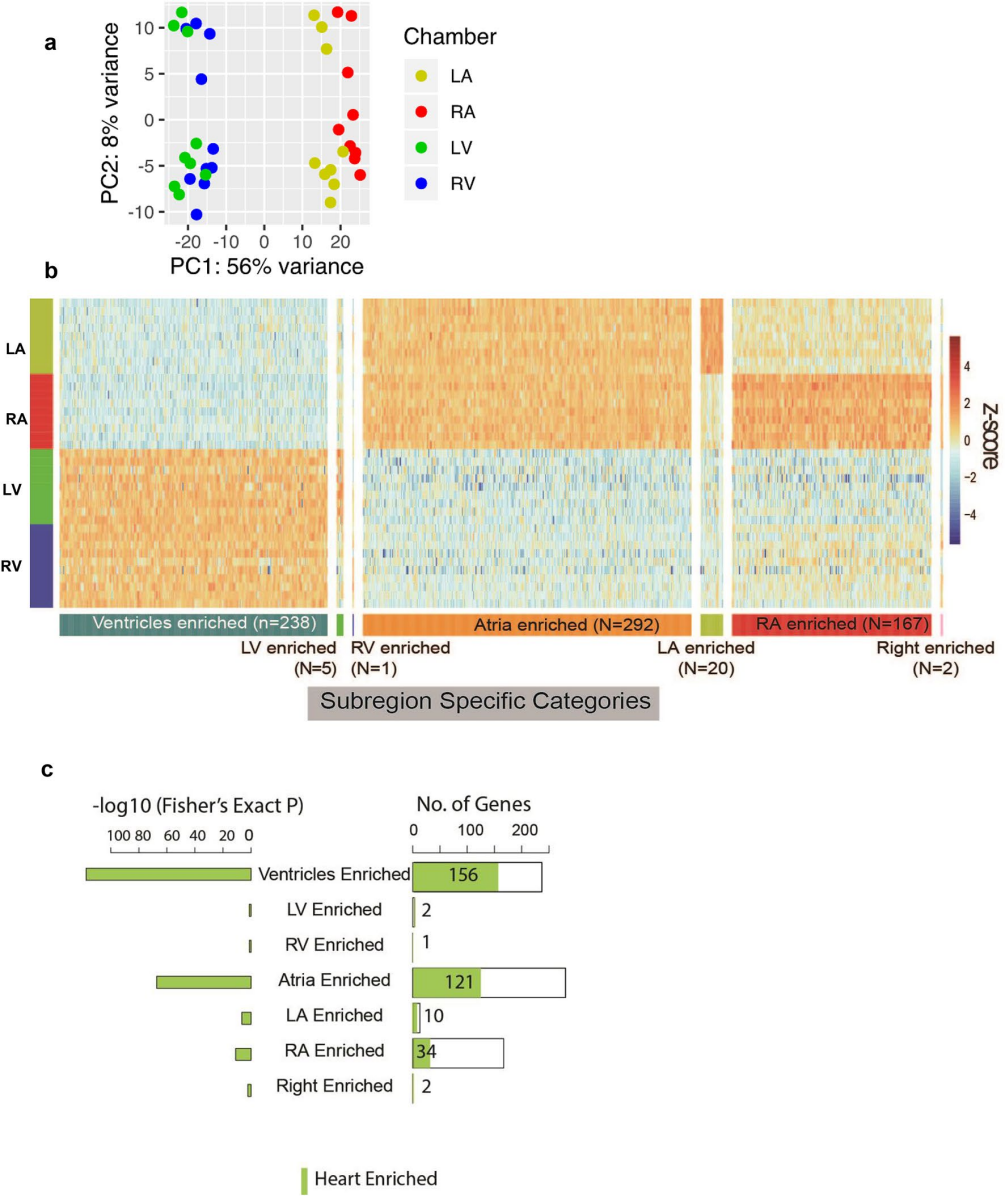
Tissue- and Subregion-Enriched genes in non-failing hearts. (**a**) The two-dimensional principal subspace for all samples from non-failing hearts based on log-transformed mRNA expression FPKM. (**b)** Heatmap for scaled expression of chamber enriched mRNAs. Rows represent individual non-failing heart samples from left atrium (LA), left ventricle (LV), right atrium (RA) and right ventricle (RV) of 10 subjects, and columns represent genes ordered by regional expression enrichment. **(c)** Composition of Subregion-Enriched genes by Heart-Enriched genes.

We first focused on characterizing the NF heart RNA-Seq expression profile, with the goal of defining baseline expression differences and identifying genes that are the most relevant to heart biology and thus potential dysfunction. The heart chambers exhibited distinct transcriptional profiles, especially between atria and ventricles (Fig. 2a,b). We established eight subclasses of Subregion-Enriched genes: four for individual chambers (LV-Enriched, RV-Enriched, LA-Enriched, RA-Enriched) and four along morphological axes (Atria-Enriched, Ventricles-Enriched, Left-Enriched, and Right-Enriched) (Fig. 2b). The chamber-enrichment classification identified genes upregulated at least twofold (Benjamini-Hochberg (BH) corrected *p* < 0.01) in all pairwise comparisons with the remaining chambers. Similarly, genes enriched along a morphological axis were defined as twofold upregulated in each of the valid pairwise chamber-specific comparisons, e.g. a Ventricles-Enriched gene was at least twofold enriched in the LV versus LA, RV versus LA, LV versus RA, and RV versus RA pairwise comparisons (Supplemental Methods and Supplemental Tables 2, 3).

Genes with strong expression in heart and limited expression in other tissues can potentially serve as heart biomarkers or potential cardiovascular therapeutic targets with less off-target toxicity. Thus, we also identified a set of 4,137 Heart-Enriched genes using tissue expression data from the GTEx body map RNA-Seq^22^ and criteria that heart be among the three tissues with highest expression and have a minimum normalized FPKM of 1 (normalization details in “Methods” section).

Having established lists of both Subregion-Enriched and Heart-Enriched genes (Supplemental Table 3), we then evaluated their distribution. Subregion-Enriched genes were abundant in ventricles (238 genes) and atria (292 genes), but among specific chambers were largely confined to RA (167 genes; Fig. 2b). This finding is concordant with the strong separation between ventricle and atrium samples in the principal component analysis, and the distinct grouping of RA samples compared to LA (Fig. 2a).

Cross-comparison of the Subregion-Enriched and Heart-Enriched genes revealed significant overlap. Nearly 40% of Ventricles-Enriched and Atria-Enriched genes were also Heart-Enriched (Atria Fisher’s Exact *P* = 5.14E−68, Ventricles Fisher’s Exact *P* = 2.44E−118, RA Fisher’s Exact *P* = 1.22E−11, Fig. 2c). We hypothesized that many of these genes would play specialized roles supporting normal physiological function and have enhanced relevance to cardiovascular disease pathogenesis.

### Heart tissue expression landscape at cellular level

**Figure 3:**
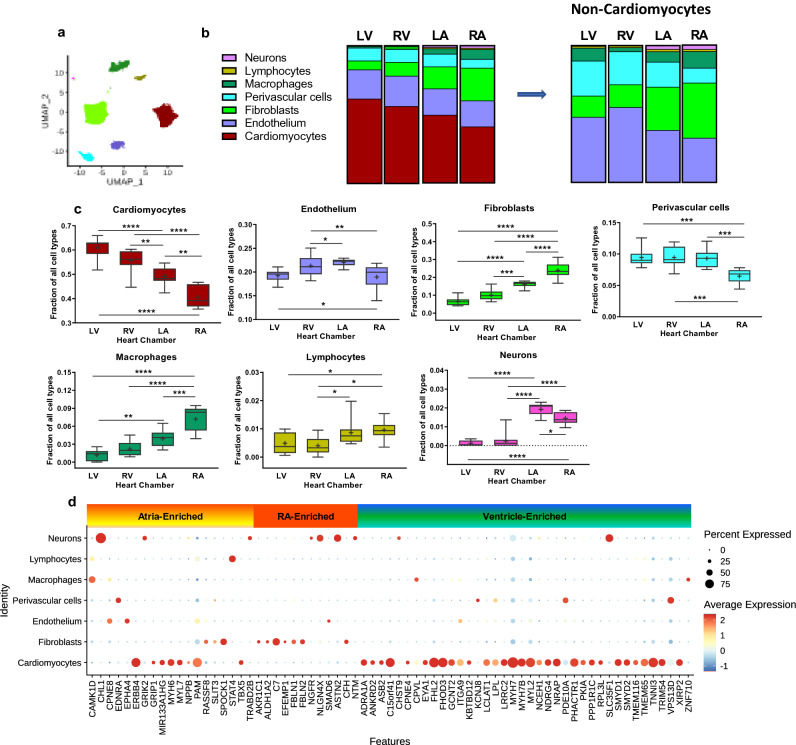
Non-failing human hearts display a regional and cellular specific transcriptome landscape. (**a**) UMAP (Uniform Manifold Approximation and Projection) projection of major cell types in NF human heart identified by single nucleus RNA-Seq (n = 7535 nuclei). Each dot represents a single nucleus, different cell type clusters are color-coded. (**b**) Cellular composition (“part-of-whole” bars) and (**c**) fractions of each cell type in each chamber of human non-failing hearts (box plots) by SCDC deconvolution analysis. Asterisks indicate the significance between the chambers by one way ANOVA followed by Tukey post-test (*p < 0.05, **p < 0.01, ***p < 0.005, ****p < 0.001) (**d**) Cell-type-specific gene expression distribution of Heart and Subregion-Enriched genes in non-failing heart. The size of the dot indicates the percentage of cells with at least one transcript detected and the color of dot represents the scaled average expression level of expressing cells.

To generate the data necessary for cell composition deconvolution, we performed single nucleus RNA-Seq (snRNA-Seq) on left ventricles from four NF and one HF donor hearts (see “Methods” section and Supplemental Table 1). After filtering, we recovered 11,716 nuclei and identified seven major cell types within human heart tissue—cardiomyocytes, fibroblasts, endothelium, macrophages, lymphocytes, perivascular cells and neurons (cardiac). Marker genes enriched in each major cardiac cell type in the single nucleus RNA-Seq dataset are shown in Supplemental Table 4. This single nucleus transcriptional profile served as a reference for the interrogation of gene expression changes at the cellular level (Fig. 3a and Supplemental Fig. 1).

Bulk RNA-Seq signal is a multiplication of average expression signal in each cell type with proportions of all cell types in the tissue. We leveraged cell type specific expression from single cell references to estimate the relative proportions of the cardiac cell types using SCDC^23^ R package by Dong et.al. (Fig. 3b). Cardiomyocytes accounted for the majority of cardiac mRNA across all chambers (61% in LV, 55% in RV, 49% in LA and 41% ion RA), followed by endothelium (19–22% of cells in each chamber), fibroblasts (6–10% in ventricles, 16% in LA and 24% RA), perivascular cells (7–9% across the chambers), neurons (0.1% in ventricles, 1.5% in LA and 2% of cells in RA) and other cell types (0.5–1%, primarily macrophages and lymphocytes). Consistent with bulk RNA-Seq inter-chamber comparisons, cellular composition in ventricles was similar (Fig. 3b,c). At the same time the representation of cardiomyocytes and fibroblasts differed between ventricles and atria: ventricles demonstrated higher cardiomyocyte proportions, whereas proportion of fibroblasts was higher in atria. Furthermore, cellular composition of right atrium was represented by the highest proportion of fibroblasts, macrophages and cardiac neurons, and the lowest cardiomyocytes representation. Additionally, we performed gene expression analysis of well-known cardiac cell type specific markers using bulk RNA-Seq data and achieved high concordance with the estimated cell proportion across chambers (Supplemental Fig. 2a).

Within non-cardiomyocyte cell population (Fig. 3b) the percentages of endothelial cells (47–55% in ventricles vs. 32–39% in atria) and perivascular cells (24–26% in ventricles vs. 11–18% in atria) were higher in ventricles, whereas fibroblast and neuron populations were dramatically higher in atria (32–40% for fibroblasts and 3% for neurons) compared to ventricles (15–16% for fibroblasts and 0.5% for neurons). Notably, the percentage of macrophages was approximately threefold higher in LV relative to RV (9.5% vs. 3%, respectively).

Consistent with previously reported snRNA-Seq experiments^11,13^, ventricles and atria exhibited distinct cell-type-specific gene expression profiles (Fig. 3d and Supplemental Fig. 1e; Supplemental Tables 3 and 4). Heart- and Subregion-Enriched cardiomyocyte markers *FHL2, FHOD3, MYH7, MYH7B, MYL2* and *TNNI3* demonstrated higher expression in ventricles, whereas *ERBB4, MYH6, MYL7* and *NPPB* genes were more enriched in atria. The restrictive expression pattern of myosin during heart development is essential for adult heart function^24^, and this notion is supported by our findings that *MYL2* is more enriched in ventricles whereas *MYL7* is more enriched in atria.

### Linking NF Heart- and Subregion-Enriched genes with biological function and disease association

**Figure 4:**
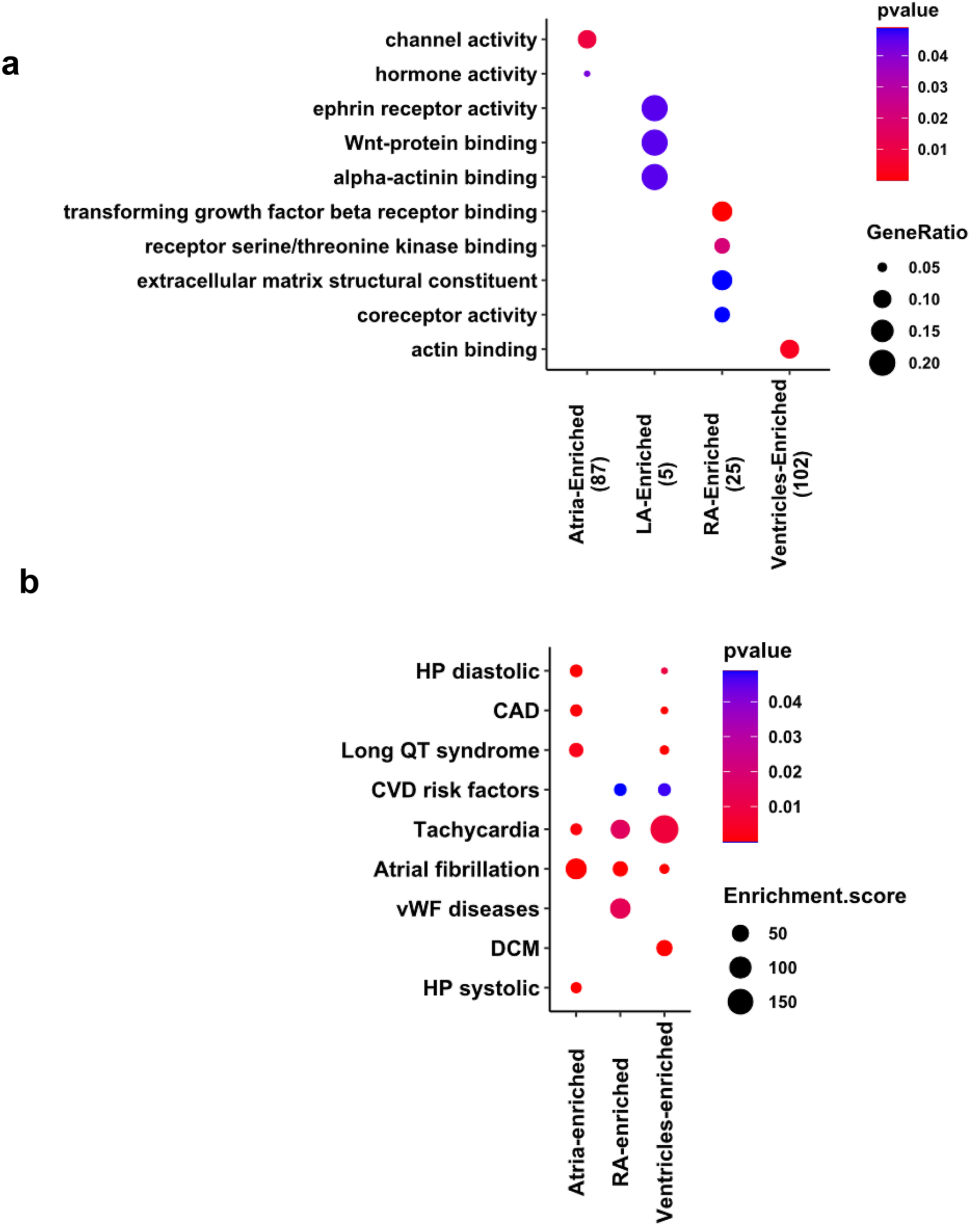
Non-failing human hearts demonstrated chamber enriched pathways and chamber specific genes associated with cardiovascular disorders. (**a**) Comparison of over-representative GO (Gene Ontology) pathways among Subregion-Enriched gene sets (sets with fewer than five genes are omitted). Gene Ontology pathways summarizing the top 10 significantly enriched terms (adjusted *p* value < 0.05) were shown. The size of the dot reflects the GeneRatio (Proportion of Subregion-Enriched genes that associated with the given GO) and the color of the dot indicates increasing significance of the enriched pathways from blue to red. Number of genes in the gene sets are in parentheses. (**b**) Significantly enriched GWAS archived cardiovascular diseases represented by heart abundant chamber specific genes. The size of the dot reflects the Enrichment score (the odds that the GWAS trait is over-represented among genes of interests vs. the odds among all other genes) and the color of the dot indicates increasing significance of the enriched GWAS trait from blue to red. Significantly enriched terms are presented and significance is indicated as *p* < 0.05.

The Subregion-Enriched gene sets showed distinct gene ontology biological processes (Fig. 4a), suggesting potential sub-functionalization. Atria-Enriched genes are associated with ion channel activity and hormone activity, reflecting the role of atria as an endocrine and electrical signaling center of the heart. Actin binding genes (*FHOD3*, *MYH7*, *MYH7B*, *MYL2*, *MYL3*, *NRAP*, *PHACTR1*, *TNNC1*, *TNNI3*, *TWF2*, *XIRP2*), which are involved in the formation and disassembly of actin filament and cardiomyocyte function, are highly enriched in ventricles. Within LA samples we observed activation of pathways responsible for ephrin receptor activity, Wnt- and alpha-actinin binding. The pathways specifically enriched in RA included TGF beta receptor binding, serine/threonine kinase receptor binding, extracellular matrix (ECM) constituent and general coreceptor activity (Fig. 4a).

Human genetics provides additional power for the interrogation of the direct gene-disease association. We cross-referenced the 333 genes (Supplemental Table 3) present in both the Heart- and Subregion-Enriched gene sets against the GWAS catalogue of heart disease-associated genes and variants^25,26^. The 156 Heart- and Ventricle-Enriched genes were associated with dilated cardiomyopathy, while the 128 Heart- and Atria-Enriched genes were highly associated with atrial fibrillation and systolic hypertension. The 41 Heart- and RA-Enriched genes were highly associated with atrial fibrillation and vWF-related diseases (Fig. 4b). The Ventricles-Enriched *MYH7, FHL2, TNNC1* and *TNNI3* genes were associated with dilated cardiomyopathy (DCM). Atria-Enriched *GJA5*, *KCNA5* and *NPPA*, RA-Enriched *HCM4*, *MIR100HG*, *REC114* and *SMAD7* and Ventricles-Enriched *KCNJ2*, *MYH7*, *PHLDB2*, *RPL2L* and *SLC35F1* were associated with atrial fibrillation. The disease association is consistent with Ventricles-Enriched genes involved in actin function, and Atria-Enriched genes related to channel activity and hormone signaling (Fig. 4a).

### HFrEF is a left heart disease with shared and distinct transcriptional changes in left atrium and left ventricle

**Figure 5:**
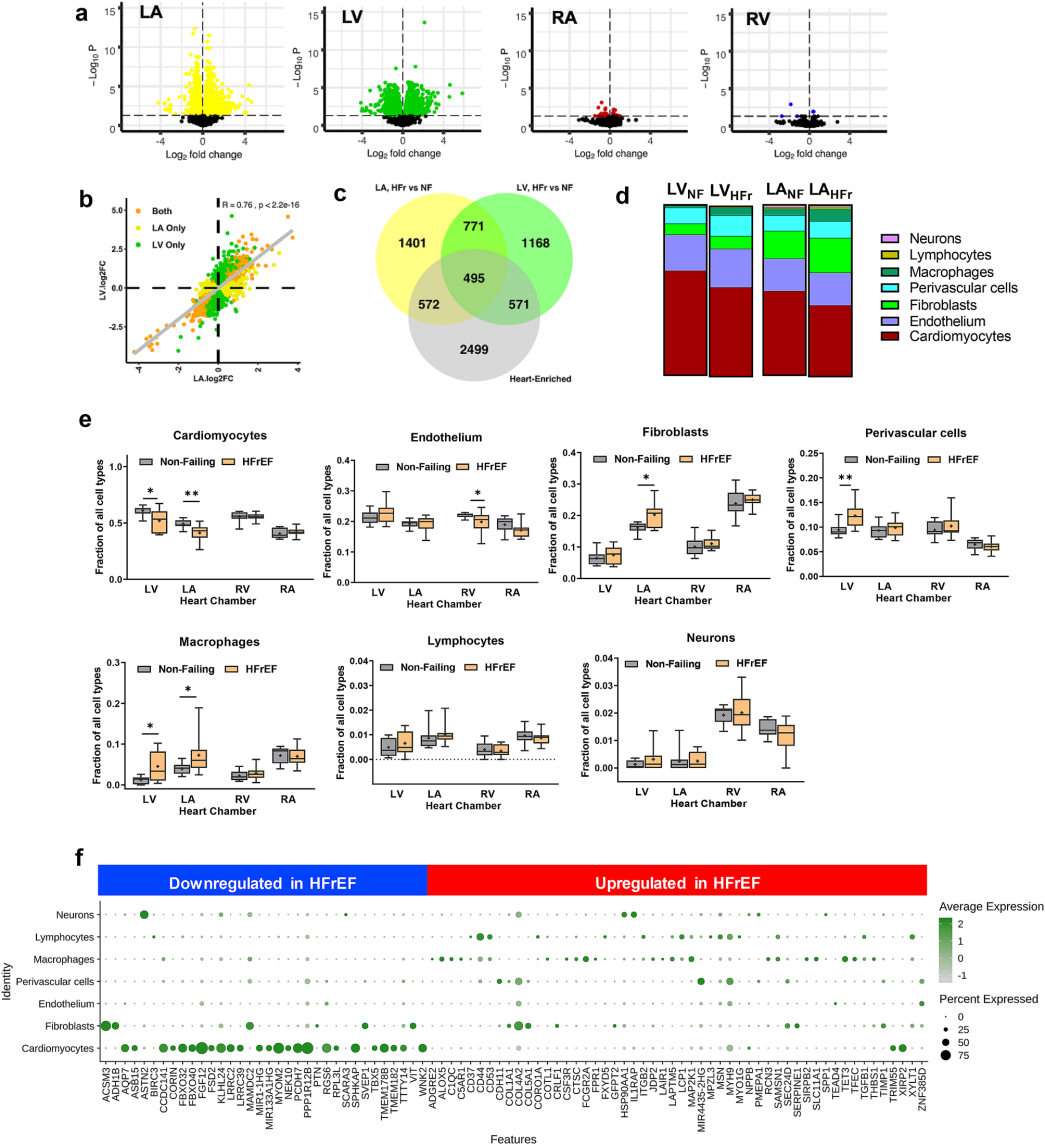
HFrEF-diseased heart displayed regional and cellular alterations in gene expression profiles. (**a**) Volcano plots represent differentially expressed genes (DEGs) in HFrEF versus Control in each heart chamber. Genes with BH adjusted *p* value < 0.05 are color-marked. (**b**) Correlation of DEG expression in LA and LV. Significantly altered DEGs in both LA and LV are marked orange, DEGs that are only significantly regulated in LA or LV are labeled as yellow and green respectively. Refer to Supplemental Table 5 for the number of genes that were classified into different regulatory groups by regulation direction in the LA and LV. (**c**) Venn diagram showing the intersection of HFrEF DE genes in the LA and LV with Heart-Enriched genes. (**d**) Averaged cellular composition (“part-of-whole” bars) and (**e**) fractions of each cell type (box plots) in the LA and LV of human non-failing (NF) and failing hearts (HFr) by SCDC deconvolution analysis. Significance between HFrEF and NF groups was evaluated by Mann Whitney test. Significant differences between the groups are indicated as exact *p* values. LV_NF_, left ventricle of non-failing heart. LV_HFr_, left ventricle of HFrEF heart. LA_NF_, left atrium of non-failing heart. LA_HFr_, left atrium of HFrEF heart. (**f**) Cell-type-specific gene expression distribution of selected Heart-Enriched DEGs (fold change less than 0.5 or more than twofold, BH adjusted *p* value < 0.01) in HFrEF left heart by single nucleus RNA-Seq. The size of dot indicates the percentage of cells with at least one transcript detected and the color of dot represents the scaled average expression level of expressing cells.

Based on principal component analysis, the largest expression variations were detected between chambers followed by, to a lesser extent, disease and gender (Supplemental Fig. 3). The small sample size limited the interpretation of gender and age as factors in the HFrEF expression profiles. When exploring chamber specific expression alterations in HFrEF, we controlled for the effect of gender and age (Methods). Comparison of the transcriptional changes in chambers of HFrEF and NF heart samples revealed (Fig. 5a) that most of the gene expression alterations occurred in HFrEF left heart, with a comparable number of dysregulated genes in LA (1553 downregulated and 1686 upregulated) and LV (1421 downregulated and 1584 upregulated). In contrast, very few genes were dysregulated in the right heart: 38 downregulated and 32 upregulated in RA, and 3 downregulated and 2 upregulated in RV (Fig. 5a, Supplemental Tables 5–7). The expression changes of differentially expressed genes (DEGs) in LA and LV are highly correlated (Fig. 5b and Supplemental Tables 5–7). Although most dysregulated genes in HFrEF left heart are chamber-specific (932 down and 1041 up in LA and 796 down and 943 up in LV), there are 615 upregulated genes and 635 downregulated genes shared by LA and LV. We have found genes that were dysregulated in opposite directions in LA and LV (LA down LV up: *FRYL, KPNA3, PAIP1, RPL5, THOC1* and *VPS29* and LA up LV down: *C16orf91, CCDC8, CLSTN3, DHCR7, MAGEF1, NTMT1, PTGIS, QTRT1, TAB1* and *TPI1*).

Genes dysregulated in the HFrEF left heart were also highly enriched for Heart-Enriched genes (32.9% overlap; Fisher’s Exact *P* < 2.2e−16; Fig. 5c), with 971 downregulated and 96 upregulated genes in LA and 838 downregulated and 228 upregulated genes in LV (Supplemental Tables 3, 5–7).

### HFrEF orchestrated gene expression alterations at the cellular level

Having established that the expression changes in HFrEF hearts were mostly present in the left heart, we next wanted to evaluate heart failure-associated changes in the predicted cell type composition within the LA and LV (Fig. 5d,e; Supplemental Fig. 2b). By mRNA content (Fig. 5e), HFrEF samples exhibited a significant decrease in cardiomyocyte proportion within LV and LA and demonstrated simultaneous downregulation of cardiomyocyte-specific genes in the left heart (*AQP7, ASB15, CCDC141, CORIN, FBXO32, FBXO40, FGF12, FSD2, KLHL24, LRRC2, LRRC39, NEK10, MYOM2, PCDH7 and PPP1R12B*) (Fig. 5f, Supplemental Fig. 4, Supplemental Tables 4, 6, and 7). The proportion of fibroblasts was only significantly increased in HFrEF LA (35%, *p* < 0.05), but the expression of collagen-related genes (*COL1A1, COL1A2, COL3A1, COL4A2, COL5A1*) and fibrosis markers (*TIMP1, FN1*, and *SERPINE1* and *SERPINE2)* were dramatically increased in both chambers of HFrEF left hearts (Fig. 5d–f, Supplemental Fig. 4, Supplemental Tables 4, 6, and 7). The cell-type-specific gene expression was significantly dysregulated and may represent different stages of cardiac fibrosis, different functional activities of fibroblast clusters, or both^27,28^ (Supplemental Fig. 4, Supplemental Tables 4, 6, and 7). Significantly increased representation of perivascular cells in LV of HFrEF (*p* < 0.05) was accompanied by at least twice the level of expression of *ADAMTS12, CD44, CDH11, DAB1* and *MYH9* genes (Fig. 5d–f, Supplemental Fig. 4, Supplemental Tables 4, 6, and 7). The proportion of macrophages in the left heart of HFrEF was significantly higher compared to NF samples (*p* < 0.05) and the genes representing the macrophage transcriptional signature (*ALOX5, C1QC, C5AR1, CD163, COTL1, CSF3R, CTSC, FCGR2A, FPR1, JDP2, LAIR1, LAPTM5, MAP2K1, RCN3, SAMSN1, SIRPB2, SLC11A1, TET3, and TFEC*) were significantly higher in the HFrEF left heart relative to NF heart (Fig. 5d–f, Supplemental Fig. 4, Supplemental Tables 4, 6, and 7). Thus, the transcriptional perturbations observed in HFrEF are likely a combination of both changes to cell composition and cell-specific transcriptional alterations.

### HFrEF left heart displayed shared and distinct signaling pathways and disease associations

**Figure 6:**
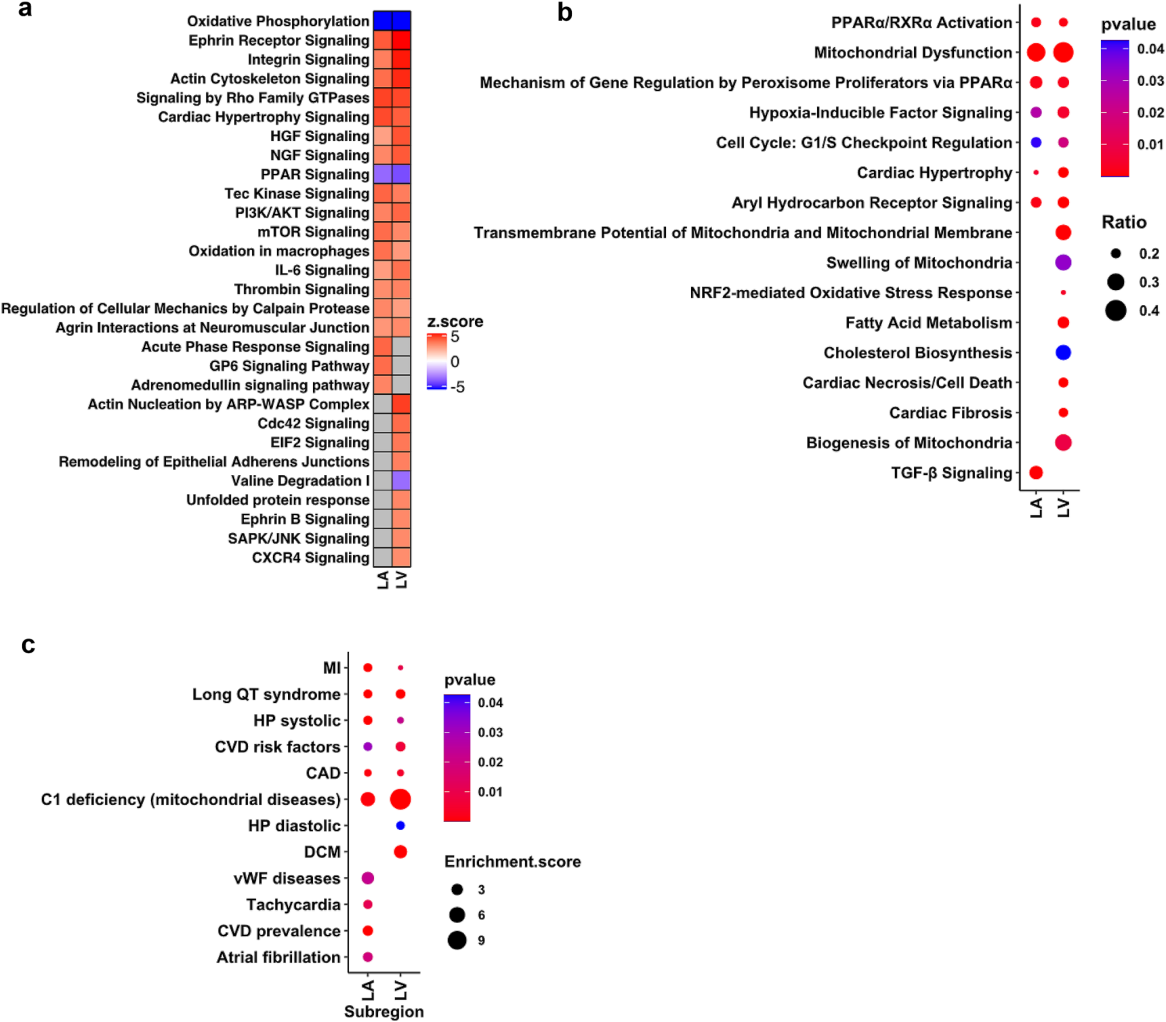
HFrEF heart displayed shared and distinct signaling pathways and disease associations. (**a**) Heatmap shows significantly overrepresented canonical pathways in HFrEF LA and LV by IPA on HFrEF DEGs (**b**) Dot plot represents enriched clinical pathology and pharmacological toxicity lists in the LA and LV by IPA on HFrEF DEGs. The size of the dot reflects the Ratio (the number of HFrEF DEGs that maps to the tox list divided by the total number of genes that map to the same tox list) and the color of the dot indicates increasing significance of the enriched pathways from blue to red. (**c**) Significantly enriched GWAS archived cardiovascular diseases represented by DEGs in HFrEF-diseased hearts. The size of the dot reflects the Enrichment score (the odds that the GWAS trait is over-represented among genes of interests vs. the odds among all other genes) and the color of the dot indicates increasing significance of the enriched GWAS trait from blue to red. Significantly enriched terms are presented and significance is indicated as *p* < 0.05.

To better understand the mechanisms of HFrEF disease pathogenesis and progression, we next focused on regional expression changes within the LA and LV and alterations in the molecular signaling pathways. Ingenuity Pathway Analysis (IPA) revealed broad activation of oxidative stress response, inflammatory signaling, Rho GTPases pathway, Ephrin receptor signaling, hypertrophy and fibrosis signaling, suppression of oxidative phosphorylation and PPAR signaling in both the LA and LV (Fig. 6a). At the same time, the LA and LV displayed uniquely altered pathways: the LA exhibited acute phase responses signaling, adrenomedullin signaling, and GP6 signaling pathways, while the LV exhibited activation of Cdc42 signaling, EIF2 signaling, and unfolded protein response, and suppression of amino acid metabolism pathways. HFrEF-associated left heart pathology has been linked to mitochondrial dysfunction, fatty acid metabolism, cardiac hypertrophy and cardiac fibrosis. Oxidative stress, mitochondrial permeability, and cardiac necrosis were more prominent in the LV. Notably, TGF-beta signaling (*ACVR1, ACVR1B, INHA, IRF7, KRAS, MAP2K1, MAP2K3, MAP3K7, MAPK13, MAPK3, NKX2-5, NRAS, PIAS4, PMEPA1, RAF1, RRAS, SERPINE1, SKI, SMAD2, SOS2, TAB1, TFE3, TGFB1, TGFB3, UBB, VDR, ZNF423*) was identified in the LA, where we observed a significant increase in fibroblasts populations, but not the LV. mRNA transcripts associated with mitochondria biogenesis (*CAV2, COX10, DNAJA3, PRDX3, PTCD2, RAB3A, TFAM*) and mitochondrial permeability (*BCL2L1, HSPA5, KRT8*) were altered in the LV but not the LA of the failing heart (Fig. 6b).

Next, we performed an analysis of the HFrEF altered genes and GWAS traits. The enrichment analysis revealed that genes dysregulated in the left heart are enriched for associations with mitochondrial diseases, Long QT syndrome, Myocardial Infarction (MI), Coronary Artery Disease (CAD), Cardiovascular Disease (CVD) risk factors and systolic hypertension. The LA subregion dysregulated genes are strongly associated with atrial fibrillation, vWF-related diseases, tachycardia, and CVD prevalence, whereas DCM associated genes are only significantly enriched in LV altered genes (Fig. 6c). Additionally, when we overlaid the HFrEF-altered genes with the cardiomyopathy testing panel (Comprehensive Cardiomyopathy NGS panel of genes with hereditary cardiomyopathies from Fulgent^29,30^), 60 out of 129 cardiomyopathies panel genes were significantly altered in HFrEF LA or LV. For example, *HCN4* is upregulated in both the LA and LV. *MYH7, MYL3* and *TNNC1* are downregulated in the LV (Supplemental Fig. 5). Notably, RAS/mitogen-activated protein kinase (MAPK) signaling pathway genes *NRAS, KRAS, RRAS* were upregulated in HFrEF left heart. Previous studies have associated this pathway with the pathogenesis and phenotypes in Noonan syndrome featured by heart defects^31,32^.

### WGCNA network analysis identified gene regulatory modules and key hub genes in HFrEF hearts

**Figure 7:**
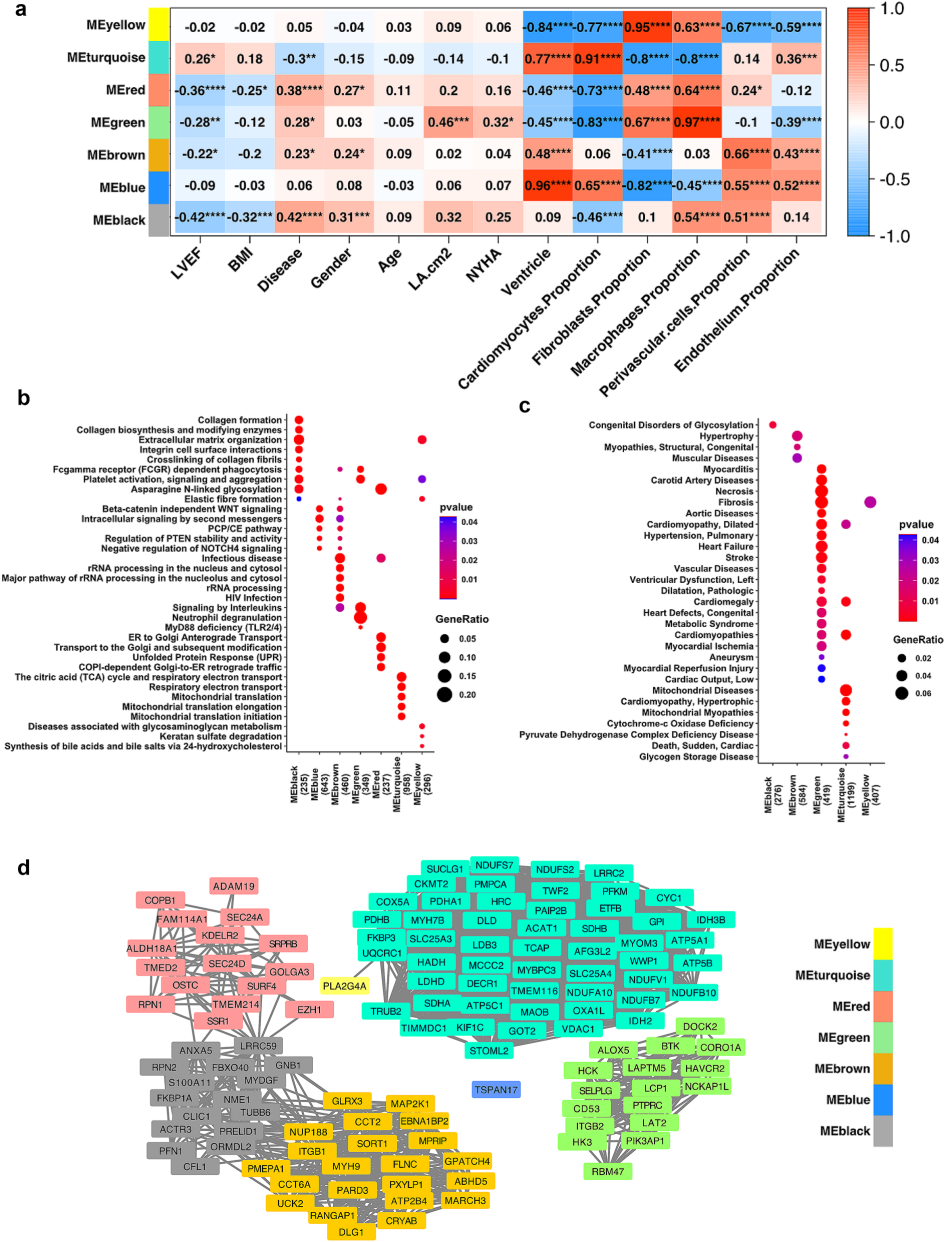
Network analysis revealed key modules and genes altered in HFrEF-diseased heart. (**a**) WGCNA analysis classified genes into 7 major modules. The heatmap shows the correlation score between each of the gene modules with the analyzed sample and clinical feature groups (*p < 0.05, **p < 0.01, ***p < 0.005, ****p < 0.001). Comparison of over-representative (**b**) Reactome pathways and (**c**) cardiometabolic diseases associated MeSH terms among modules determined by WGCNA analysis. The size of the dot reflects the GeneRatio (the proportion of genes from list of interests that maps to the pathway) and the color of the dot indicates increasing significance of the enriched pathways from blue to red. Number of chamber enriched genes that overlap with the over-representative pathways are shown in parenthesis. Significantly enriched terms are presented in (**b,c**) and significance is indicated as *p* < 0.05. (**d**) Disease-associated hub genes (*p* < 0.05) with top connectivity are visualized by Cytoscape. Color of each node corresponds with the module color in (**a**).

To further interrogate the functional gene regulatory network associated with HFrEF, we performed Weighted Gene Co-expression Network Analysis (WGCNA)^33,34^ and identified 7 distinct gene modules (11,127 genes expressed in at least one subregion or disease group from 82 human heart samples). Next, we investigated the underlying relationships between gene modules and key available clinical traits and cell compositions (Fig. 7a–c). The modules color-coded as Black, Brown, Green, and Red positively correlated with HFrEF status and negatively correlated with left ventricle ejection fraction (LVEF) and/or Body Mass Index (BMI). Module Black exhibited positive correlation with proportion of macrophages and perivascular cells and reciprocal correlation with cardiomyocytes proportion in heart tissue. Additionally, module Black contained transcripts associated with collagen formation, extracellular matrix organization, Fc-gamma receptor dependent phagocytosis and glycosylation. Module Brown demonstrated positive correlation with the expression of ventricle enriched genes and proportion of perivascular cells and endothelium, whereas the correlation with fibroblasts proportion in heart tissue was reciprocal. Module Brown transcripts were enriched with rRNA processing and interleukin signaling pathways and associated with hypertrophy and myopathies. Modules Green and Red demonstrated negative correlation with ventricular gene expression, proportion of cardiomyocytes, and positive correlation with proportion of fibroblasts and macrophages. Module Green was enriched with neutrophil degranulation associated transcripts, interleukin signaling, and a wide range of cardiovascular diseases, including heart failure, stroke, pulmonary hypertension, and myocarditis, while module Red was associated with Golgi and ER trafficking, and the unfolded protein response. Importantly, modules Green exhibited a positive correlation with LA volume, which reiterated the importance of LA function in HFrEF. Module Turquoise was negatively associated with HFrEF diagnosis and the proportion of fibroblasts and macrophages in heart tissue composition. Positive correlations within Module Turquoise included LVEF, higher ventricular gene expression, cardiomyocytes and endothelium proportion. Module Turquoise was enriched in electron transport and mitochondrial function associated transcripts, and was associated with mitochondria diseases, mitochondria myopathies, and sudden cardiac death. Furthermore, we identified 133 HFrEF significantly associated hub genes with top connectivity and observed the intermodular connection among HFrEF associated Brown, Black and Red modules (Fig. 7d). Modules Blue and Yellow were not significantly associated with HFrEF.

## Discussion

Adult human heart has a complex tissue structure, and four heart chambers demonstrate heterogeneous unique cell composition^11,13^. We found that atrial and ventricular tissues differed in their cellular composition. We and others^11,13^ have demonstrated that not only cardiomyocytes comprised higher percentage in ventricles and fibroblasts in atria, but that atrial and ventricular subset of cardiac cells demonstrated different gene expression profiles and specialized properties. For each chamber we defined a set of enriched genes involved in the progression of several cardiovascular diseases: GWAS signals for atrial fibrillation were associated with 41 RA-Enriched genes, whereas GWAS signals for dilated cardiomyopathy were associated with Ventricles-Enriched *MYH7, FHL2, TNNC1* and *TNNI3* genes. In concert with published studies that analyzed each chamber separately^8,11,13^, our results emphasize the shared functions of ion channels and hormone activities in both Atria- and Ventricles-Enriched genes involved in actin function.

Heart failure (HF) is a complex clinical syndrome characterized by deficient cardiac output that is unable to support the metabolic needs of the body. HF with reduced ejection fraction (HFrEF) represents half of all HF cases and has significant morbidity and mortality for all patients^6^. Although HFrEF can be caused by different etiologies and patients demonstrate high heterogeneity, transcriptome analysis may provide additional phenotypic readouts of underlying cell biology after injury beyond clinical symptoms. Extensive transcriptome and bioinformatics analyses allowed us to pinpoint the common gene network and signaling pathways that are shared by HFrEF patients, enhancing our understanding of underlying disease mechanism and biomarker identification. HFrEF heart samples displayed left heart specific transcriptional changes in both the LV and LA, whereas the transcriptional changes in the right heart were negligible. Such significant alterations in the transcriptional profile of LA HFrEF hearts have not previously been reported, as the majority of studies were focused on LV transcriptome analysis^15–18^. Gene expression changes and signaling pathway alterations in the LA might unmask the underlying mechanisms leading to LA dysfunction in HFrEF. HFrEF LA displays a vast transcriptional landscape shift, which is consistent with the fact that LA buffers pressure and flow oscillations during the cardiac cycle by acting as an elastic reservoir and LA dilation is closely associated with HFrEF patients’ prognosis^35–37^. The expression changes of differentially expressed genes in the LA and LV were highly correlated, with nearly one third of the dysregulated genes shared by LA and LV (Fig. 5b and Supplemental Table 5). Two thirds of the genes dysregulated in the HFrEF left heart are specific for either the LA or LV. IPA analysis demonstrated broad activation of oxidative stress response, inflammatory signaling, Rho GTPases pathway, Ephrin receptor signaling, hypertrophy and fibrosis signaling, suppression of oxidative phosphorylation and PPAR signaling in both the LA and LV (Fig. 6a) and supported the hypothesis of a shift of the homeostatic transcriptional program and cardiac function to cardiac stress signaling. In the heart failure setting, the LA exhibited activation of the acute phase response signaling, adrenomedullin signaling, and GP6 signaling pathways, while activation of Cdc42 signaling, EIF2 signaling and unfolded protein response, and suppression of amino acid metabolism pathways were documented in the LV. The major findings from our analysis of the HFrEF altered genes and GWAS traits included strong association between LA-dysregulated genes and atrial fibrillation, vWF-related diseases, tachycardia, and CVD prevalence, whereas DCM-associated genes were significantly enriched in LV altered genes. Remarkably, half of the genes present in a cardiomyopathy genetic testing panel^29,30^ are dysregulated in HFrEF left heart, which highlights the potential utility of transcriptome analysis in target and biomarker discovery.

Understanding the cell type composition and communication between cardiomyocytes and diverse non-cardiomyocytes (endothelial cells, fibroblasts, perivascular cells, immune cells and cardiac neurons) are essential for the understanding of cardiac function and disease pathogenesis. Recent advances in single cell RNA-Seq technology enable one to dissect the heart transcriptome at single cell resolution^11–13,21^. However, the relatively high cost and the potential bias introduced by the cell dissociation and library preparation steps limit its application in characterizing a large clinical cohort and accurately analyzing cell composition for certain tissues and cell types. Using the cell type reference established by single cell analysis, we performed deconvolution of bulk tissue RNA-Seq to estimate the cell compositional changes across chambers and disease status. Our observation of cell compositional changes and transcriptional program shifts in HFrEF left hearts revealed novel pathological changes in the resident cell types of heart tissue, also supported by recent reports^27,28,38–40^. Macrophage infiltration during HFrEF progression was associated with various cardiomyopathies, which could in turn induce myofibroblast differentiation and cardiac remodeling^38^. Accumulating evidence suggested pericytes could play an important role in heart failure, especially in ischemic cardiomyopathies^39,40^. Recent studies demonstrated that the detachment of pericytes from EC/pericyte communication could promote differentiation into myofibroblasts, linking perivascular cells to fibroproliferation and ECM remodeling^41,42^. In addition, increased fibroblast activity and excessive collagen and ECM composition have been shown to promote myocardial fibrosis and remodeling, and eventually heart failure^27,28^. Notably, by WGCNA network analysis, we found strong association of cell composition with subregion and disease gene expression programs. The latter sheds light on the role of cellular components and context in the cardiac transcription landscape.

Single cell deconvolution analysis allowed us to avoid the cell/nuclei dissociation bias and use the whole transcriptome to perform more accurate cell identification and classification. Thus, our approach complemented the results achieved by orthogonal methods^3,43^ (stereological and flow cytometric based methods of isolated nuclei), which might suffer from sampling and dissociation bias or the availability of cell type specific markers. Nevertheless, several limitations should still be acknowledged. The primary assumption of the deconvolution method is that the relative mRNA content across cell types in the reference dataset are comparable with those in the bulk RNA-Seq samples. This assumption might be violated, if total mRNA content varies due to changes in cell volume, discordance between cytoplasmic and nuclear levels, or alteration in metabolic activity. Such discordance could impact precision of intra-sample cell proportions as well as potentially inter-sample comparisons. For example, the observed overestimation of cardiomyocytes is likely due to the large cell size and more transcriptionally active state. Despite such limitation, we still see value in providing the deconvolution results so that future publications can incorporate our findings into orthogonal datasets. Additionally, to achieve accurate deconvolution, it is critical to assure that the reference dataset adequately captures the range of cell types and transcriptional states present in the bulk RNA-seq samples, since missing cell types in reference could skew the cell proportions calculation. In the manuscript, we have focused on the characterization of major cardiac tissue cell types keeping in mind that many genes fundamental to major cell identity are unlikely to change dramatically in the disease state. However, we couldn’t drill down to more detailed subtypes of cells, for instance, the disease activated fibroblasts. This will rely on future single cell/nucleus analysis on failing hearts similar to analyses done in healthy hearts by recent studies^11,13^. Furthermore, since the estimates are presented as relative proportions of the cells, we can only speculate as to whether reported changes in gene expression are driven by decreased cardiomyocyte number, alteration in cell size and transcription activity, or even by increased presence of cell types other than cardiomyocytes.

## Conclusion

The deep exploration of the regional and cellular transcriptomic landscape in non-failing and HFrEF human hearts has expanded our understanding of the molecular basis of heart failure pathogenesis. We have delineated the Heart- and Subregion-Enriched gene signatures in association with cardiac function and disease specification. The dramatic HFrEF-associated transcriptomic changes in the left heart suggested that the restoration of LA function may be equally important as the improvement and rescue of LV function. We have elucidated hub genes and key pathways related to HFrEF, which shed light on previous unknown or neglected targets. Our study suggested that in depth exploration of fibroblast state transition, pericyte-fibrosis link and macrophage infiltration would provide better understanding of HFrEF etiology/progression and support discovery of novel biomarkers and therapeutic targets. Lastly, the link between HFrEF dysregulated genes and cardiometabolic disease associated genes reveal the power of combining deep expression profiling and human genetics in target and biomarker discovery.

## Methods

### Tissue collection

All human specimens were collected under Institutional Review Board or Independent Ethics Committee approval with appropriate informed consent in compliance with all applicable laws and regulations. The consent procedure and study design process are governed by the master agreements between Amgen and the commercial entities Zenas, AnaBios, and IIAM. In all cases, materials obtained were surplus to clinical practice standard of care. Patient identity and PHI/identifying information were redacted from tissues and clinical data prior to submission to Amgen. Human tissue specimens were obtained from the following institutions:

- Zenas Technologies—Metairie, LA—Tissue collection was approved by Tulane University Human Research Protection Office & IRBs (HRPO) and obtained through a retrospective study conducted in 2014–2015. The postmortem interval for all donors was reported to be within 3 h (Supplemental Methods, Supplemental Table 1).
- AnaBios Corporation—San Diego, CA—All human hearts that were used for this study were obtained by legal consent from organ donors in the US. Policies for donor screening and consent are the ones established by the United Network for Organ Sharing. Organizations supplying human tissues to AnaBios follow the standards and procedures established by the US Centers for Disease Control and are inspected biannually by the Department of Health and Human Services. Tissue distribution is governed by internal IRB procedures and compliance with HIPAA regulations regarding patient privacy. All transfers of donor organs to AnaBios are fully traceable and periodically reviewed by US Federal authorities. AnaBios conducted a custom prospective collection in 2016. The warm ischemic time (WIT) was less than 1 h (Supplemental Methods, Supplemental Table 1).
- International Institute for the Advancement of Medicine (IIAM)—Edison, NJ—IIAM provided non-transplantable hearts from both non-failing and heart failure donors. Tissue collection was approved by IIAM’s External Review Committee (ERC). The warm ischemic time (WIT) was less than 1 h (Supplemental Methods, Supplemental Table 1).

Bulk RNA-Seq were performed on 10 Control non-failing (NF) and 12 HFrEF heart samples collected from the left atrium, left ventricle, right atrium and right ventricle obtained from Zenas Technologies (Supplemental Table 1). Single-nucleus RNA-Seq was performed on 4 NF left ventricle heart tissue samples were obtained from AnaBios Corporation (n = 3) and IIAM (n = 1) and one HF sample from IIAM (Supplemental Table 1). All the tissue samples were frozen in liquid N2 and stored at -80C.

### RNA isolation and sequencing analysis of the heart

Frozen heart tissue samples from NF (9 LA, 9 LV, 9 RA and 10 RV) and HFrEF (12 LA, 12 LV, 12 RA and 11 RV) sets (Zenas Technologies, Metairie, LA) were subjected to dry pulverization (Supplemental Methods). RNA extraction was performed using the RNeasy Micro Kit (Qiagen, USA) with on-column DNase treatment (Qiagen, USA) according to the manufacturer’s instructions. RNA concentration and integrity were assessed using a NanoDrop 8000 (Thermo Fisher, USA) and a Bioanalyzer (Agilent, USA). Samples with ≥ 100 ng total RNA and RNA integrity numbers (RIN) ≥ 7 were used for sequencing. Total RNA (100 or 500 ng) was used for cDNA library preparation using a modified protocol based on the Illumina Truseq RNA sample preparation kit and the published method for strand-specific RNA-Seq^44,45^. The enriched cDNA libraries were analyzed in an Agilent Bioanalyser and quantified by Quant-iT™ Pico-Green assays (Life Technologies, USA) before being loaded onto the HiSeq platform (Illumina, USA). RNA-Seq sequencing reads were aligned using OSA aligner^46^ embedded in v10.0 of the Omicsoft Array Suite (Omicsoft, USA) based on human genome version GRCh38 and gene model GENCODE v24. Quantification was performed to the gene level based on Omicsoft implementation of RSEM^47^. Gene expression was represented as normalized fragments per kilobase per million reads (FPKM). FPKM values were normalized with a refinement of the commonly employed upper-quartile method^48^ that sets the FPKM to a value of 10 at the 70th percentile^49,50^. 2D Principal component analysis (PCA) plots based on the log transformed FPKM value of all genes with average read counts > 50 for all samples were created to characterize the trends exhibited by the expression profiles of samples across heart subregions and disease groups. Lowly expressed genes having fewer than five samples at normalized FPKM > = 1 were removed from pairwise comparisons. Raw reads counts from the expressed genes were compared between different heart chambers or disease groups using R package DESeq2 (v1.26.0) following Negative Binomial distribution^51^.

### Heart tissue specificity and subregion enrichment calculation

Heart tissue specificity was calculated using RNA-Seq of 30 different normal tissues in the GTEx database^22^ (Supplemental Methods). For heart chambers obtained for this study, six pairwise comparisons were performed between any two. Genes with BH corrected *p* value < 0.01 and Fold Change > = 2 or < = 0.5 were selected as differentially expressed genes between subregions of the heart. A stringent protocol was applied to select chamber enriched genes (Supplemental Table 2, Supplemental Methods). Chamberenriched genes were classified into categories: (1) Single heart chamber enriched in either LA, RA, LV or RV; (2) Enriched in both Atria or Ventricles; (3) Enriched in left or right heart (Supplemental Table 3).

### Pathway analysis

Gene expression that was different in heart subregions or disease groups was visualized in a graphic heatmap using the ComplexHeatmap (v2.2.0) R package^52^. The DEGs were further annotated by Ingenuity Pathway Analysis (IPA; QIAGEN, USA) on canonical pathways and toxicity lists. Gene expression enrichment in Gene Ontology (GO)^53,54^, Reactome^55^ and Disease Medical Subject Headings (MeSH) terms was analyzed by using clusterProfiler (v3.14.3)^56^ , ReactomePA (v1.30.0)^57^ and the meshes library (v1.12.0)^58,59^. GWAS cardiovascular terms enrichment analyses were performed with genetic associations from STOPGAP^25^ by Fisher’s exact test.

### Single-nucleus RNA-Seq (snRNA-Seq) and deconvolution analysis

snRNA-Seq was performed on left ventricle samples from 4 NF and 1 HF donor (Supplemental Table 1, Supplemental Methods) using 10X Genomics Single cell Gene Expression 3’ V3 chemistry (Cat. 1,000,075). Briefly, five 10 µm curls cut from OCT embedded human cardiac tissue were homogenized using a Dounce homogenizer with lysis buffer. The nuclei were filtered, centrifuged, washed and then resuspended in Nuclei Wash/Resuspension Buffer. DRAQ5 + nuclei were sorted and loaded into the 10 × Genomics Chromium Controller for single nucleus microfluidic encapsulation and barcoding following manufacturer’s instructions. Libraries were prepared and sequenced on NovaSeq S4 flowcell. We used cell ranger (V3.1.0) mkref package to create a “pre-mRNA” reference with pre-built GRCh38 reference package, then we used cell ranger count pipeline to align the fastq reads and generated QC matrix and count matrix^60^. The Count matrix was further analyzed with Seurat (v3.2.0) R package^61^ for batch effect correction by canonical correlation analysis, filtering, normalization, variable features identification and dimensional reduction by Principal Component Analysis (PCA). The top 30 PCAs were used in graph-based clustering based on Louvain with resolution at 1.2, and cluster specific marker genes were identified with FindAllMarkers function by Wilcoxon Rank Sum test in Seurat, cell types were determined by cross-referencing with well-established cell type markers in literature^11,13^. Uniform Manifold Approximation and Project (UMAP) was used to visualize the high dimensional cell cluster distribution.

Using single cell / nucleus RNA-Seq data of heart as references, we used SCDC (v0.0.0.9000)^23^ to perform deconvolution analysis to estimate cell type proportions in bulk RNA-seq data on 84 samples. To generate robust cell clustering for deconvolution, we performed further quality control procedure by using ‘SCDC-qc()’ function to remove cells with questionable cell-type assignments on single cell reference datasets. To integrate the deconvolution results from multiple single cell reference datasets (unpublished snRNA-seq data from NF samples and HF sample, published heart snRNA-Seq data from Broad Institute’s Single Cell Portal^11^, published heart snRNA-Seq and scRNA-Seq from www.heartcellatlas.org^13^), we used the ‘SCDC_ENSEMBLE()’ function in the SCDC R package^23^ with default settings. We constrained our cell type composition estimate on 7 major cardiac cell types classified by single cell analysis (Cardiomyocytes, Endothelium, Fibroblasts, Macrophages, Perivascular cells, Lymphocytes, and Cardiac Neuron). The differences in relative cell proportion across different chambers and disease status were visualized as bar graphs (GraphPad Prism 8) and the significance of the differences was analyzed by one way ANOVA followed by Tukey post-test across the chambers of non-failing hearts and Mann Whitney Test for the significance between HFrEF versus NF.

### WGCNA network analysis

Weighted gene co-expression network was constructed with WGCNA (v1.70–3) R package^33,34^. Briefly, 11,127 genes with median expression in any chamber and disease combination more than 1 FPKM were used in the analysis, samples were clustered based on gene expression and two outliers were filtered out. The R function pickSoftThreshold was used to obtain the optimal soft thresholding power at 16, to which adjacency is calculated based on connectivity. Hierarchical clustering and dynamic tree cut function were applied with minModuleSize as 100 to identify gene co-expression modules. Module eigengenes were calculated and correlation with sample and clinical traits were analyzed. Furthermore, intramodular analysis was performed to identify genes significantly associated with disease trait (HFrEF) within each module. We further marked genes with top connectivity (top 5%) as hub genes for each module. Network of hub genes significantly associated with HFrEF was visualized by cytoscape_v3.7.1^62^.

## Supplementary Information

Supplementary Information 1.

Supplementary Information 2.

Supplementary Information 3.

Supplementary Information 4.

Supplementary Information 5.

## Data Availability

The datasets generated in the study were deposited in the GEO database: GSE161473 is the reference Series for the publication.

## References

[1] Perbellini F, Watson SA, Bardi I, Terracciano CM (2018). Heterocellularity and cellular cross-talk in the cardiovascular system. Front. Cardiovasc. Med..

[2] Kloesel B, DiNardo JA, Body SC (2016). Cardiac embryology and molecular mechanisms of congenital heart disease: a primer for anesthesiologists. Anesth. Analg..

[3] Zhou P, Pu WT (2016). Recounting cardiac cellular composition. Circ. Res..

[4] Yancy CW (2013). 2013 ACCF/AHA guideline for the management of heart failure: executive summary: a report of the American College of Cardiology Foundation/American Heart Association Task Force on practice guidelines. Circulation.

[5] Benjamin EJ (2017). Heart disease and stroke statistics-2017 update: a report from the american heart association. Circulation.

[6] Shah KS (2017). Heart failure with preserved, borderline, and reduced ejection fraction: 5-year outcomes. J. Am. Coll. Cardiol..

[7] Asp J, Synnergren J, Jonsson M, Dellgren G, Jeppsson A (2012). Comparison of human cardiac gene expression profiles in paired samples of right atrium and left ventricle collected in vivo. Physiol. Genomics.

[8] Johnson EK, Matkovich SJ, Nerbonne JM (2018). Regional differences in mRNA and lncRNA expression profiles in non-failing human atria and ventricles. Sci. Rep..

[9] Doll S (2017). Region and cell-type resolved quantitative proteomic map of the human heart. Nat. Commun..

[10] van Heesch S (2019). The translational landscape of the human heart. Cell.

[11] Tucker NR (2020). Transcriptional and cellular diversity of the human heart. Circulation.

[12] Wang L (2020). Single-cell reconstruction of the adult human heart during heart failure and recovery reveals the cellular landscape underlying cardiac function. Nat. Cell Biol..

[13] Litvinukova M (2020). Cells of the adult human heart. Nature.

[14] Kaab S (2004). Global gene expression in human myocardium-oligonucleotide microarray analysis of regional diversity and transcriptional regulation in heart failure. J. Mol. Med. (Berl).

[15] Tan FL (2002). The gene expression fingerprint of human heart failure. Proc. Natl. Acad. Sci. U S A.

[16] Kittleson MM (2005). Gene expression analysis of ischemic and nonischemic cardiomyopathy: shared and distinct genes in the development of heart failure. Physiol. Genomics.

[17] Liu Y (2015). RNA-Seq identifies novel myocardial gene expression signatures of heart failure. Genomics.

[18] Sweet ME (2018). Transcriptome analysis of human heart failure reveals dysregulated cell adhesion in dilated cardiomyopathy and activated immune pathways in ischemic heart failure. BMC Genomics.

[19] Melenovsky V (2015). Left atrial remodeling and function in advanced heart failure with preserved or reduced ejection fraction. Circ. Heart Fail.

[20] Zhu N (2019). Left atrial diameter in heart failure with left ventricular preserved, mid-range, and reduced ejection fraction. Medicine.

[21] Cui Y (2019). Single-cell transcriptome analysis maps the developmental track of the human heart. Cell. Rep..

[22] Consortium GT (2013). The genotype-tissue expression (GTEx) project. Nat. Genet..

[23] Dong M (2020). SCDC: bulk gene expression deconvolution by multiple single-cell RNA sequencing references. Brief Bioinform..

[24] England J, Loughna S (2013). Heavy and light roles: myosin in the morphogenesis of the heart. Cell Mol. Life Sci..

[25] Shen J, Song K, Slater AJ, Ferrero E, Nelson MR (2017). STOPGAP: a database for systematic target opportunity assessment by genetic association predictions. Bioinformatics.

[26] MacArthur J (2017). The new NHGRI-EBI Catalog of published genome-wide association studies (GWAS Catalog). Nucl. Acids Res..

[27] Fu X (2018). Specialized fibroblast differentiated states underlie scar formation in the infarcted mouse heart. J. Clin. Invest..

[28] Forte E (2020). Dynamic interstitial cell response during myocardial infarction predicts resilience to rupture in genetically diverse mice. Cell. Rep..

[29] Beckmann BM, Pfeufer A, Kaab S (2011). Inherited cardiac arrhythmias: diagnosis, treatment, and prevention. Dtsch. Arztebl. Int..

[30] Kimura A (2016). Molecular genetics and pathogenesis of cardiomyopathy. J. Hum. Genet..

[31] Aoki Y, Niihori T, Inoue S, Matsubara Y (2016). Recent advances in RASopathies. J. Hum. Genet..

[32] Tidyman WE, Rauen KA (2016). Pathogenetics of the RASopathies. Hum. Mol. Genet..

[33] Langfelder P, Horvath S (2008). WGCNA: an R package for weighted correlation network analysis. BMC Bioinform..

[34] Langfelder, P. & Horvath, S. Fast R functions for robust correlations and hierarchical clustering. *J. Stat. Softw.* **46** (2012). PMC3465711 23050260

[35] Carluccio E (2018). Left atrial reservoir function and outcome in heart failure with reduced ejection fraction. Circ. Cardiovasc. Imaging.

[36] Ersboll M, Moller JE (2018). Left atrial function in heart failure with reduced ejection fraction. Circ. Cardiovasc. Imaging.

[37] Malagoli A (2019). Left atrial function predicts cardiovascular events in patients with chronic heart failure with reduced ejection fraction. J. Am. Soc. Echocardiogr..

[38] Lavine KJ (2018). The macrophage in cardiac homeostasis and disease: JACC macrophage in CVD series (Part 4). J. Am. Coll. Cardiol..

[39] Chen CW (2013). Human pericytes for ischemic heart repair. Stem Cells.

[40] Avolio E, Madeddu P (2016). Discovering cardiac pericyte biology: From physiopathological mechanisms to potential therapeutic applications in ischemic heart disease. Vasc. Pharmacol..

[41] Wang N (2017). Novel mechanism of the pericyte-myofibroblast transition in renal interstitial fibrosis: core fucosylation regulation. Sci. Rep..

[42] Sava P (2017). Human pericytes adopt myofibroblast properties in the microenvironment of the IPF lung. JCI Insight.

[43] Pinto AR (2016). Revisiting cardiac cellular composition. Circ. Res..

[44] Sultan M (2012). A simple strand-specific RNA-Seq library preparation protocol combining the Illumina TruSeq RNA and the dUTP methods. Biochem. Biophys. Res. Commun..

[45] Parkhomchuk D (2009). Transcriptome analysis by strand-specific sequencing of complementary DNA. Nucl. Acids Res..

[46] Hu J, Ge H, Newman M, Liu K (2012). OSA: a fast and accurate alignment tool for RNA-Seq. Bioinformatics.

[47] Li B, Dewey CN (2011). RSEM: accurate transcript quantification from RNA-Seq data with or without a reference genome. BMC Bioinform..

[48] Robinson MD, Oshlack A (2010). A scaling normalization method for differential expression analysis of RNA-seq data. Genome Biol..

[49] Aisenberg WH (2016). Defining an olfactory receptor function in airway smooth muscle cells. Sci. Rep..

[50] Miner K (2019). Drug repurposing: the anthelmintics niclosamide and nitazoxanide are potent TMEM16A antagonists that fully bronchodilate airways. Front. Pharmacol..

[51] Love MI, Huber W, Anders S (2014). Moderated estimation of fold change and dispersion for RNA-seq data with DESeq2. Genome Biol..

[52] Gu Z, Eils R, Schlesner M (2016). Complex heatmaps reveal patterns and correlations in multidimensional genomic data. Bioinformatics.

[53] Ashburner M (2000). Gene ontology: tool for the unification of biology. The Gene Ontology Consortium. Nat. Genet..

[54] The Gene Ontology, C. The Gene Ontology Resource: 20 years and still GOing strong. *Nucl. Acids Res.* **47**, D330–D338, 10.1093/nar/gky1055 (2019). 10.1093/nar/gky1055 PMC6323945 30395331

[55] Jassal B (2020). The reactome pathway knowledgebase. Nucl. Acids Res..

[56] Yu G, Wang LG, Han Y, He QY (2012). clusterProfiler: an R package for comparing biological themes among gene clusters. OMICS.

[57] Yu G, He QY (2016). ReactomePA: an R/Bioconductor package for reactome pathway analysis and visualization. Mol. Biosyst..

[58] Tsuyuzaki K (2015). MeSH ORA framework: R/Bioconductor packages to support MeSH over-representation analysis. BMC Bioinform..

[59] Yu G (2018). Using meshes for MeSH term enrichment and semantic analyses. Bioinformatics.

[60] Zheng GX (2017). Massively parallel digital transcriptional profiling of single cells. Nat. Commun..

[61] Stuart T (2019). Comprehensive integration of single-cell data. Cell.

[62] Shannon P (2003). Cytoscape: a software environment for integrated models of biomolecular interaction networks. Genome Res..

