# Supplementary material for: Chamber-enriched gene expression profiles in failing human hearts with reduced ejection fraction
Source: Sci Rep. 2021 Jun 4;11:11839. doi: 10.1038/s41598-021-91214-2 (PMC8178406; doi:10.1038/s41598-021-91214-2)

Supplemental Appendix

Chamber-enriched gene expression profiles in failing human hearts with reduced ejection fraction

Xin Luo[1], Jun Yin[1], Denise Dwyer[2], Tracy Yamawaki[1], Hong Zhou[1], Hongfei Ge[3], Chun-Ya Han[2], Artem Shkumatov[4], Karen Snyder[5], Brandon Ason[3], Chi-Ming Li[1], Oliver Homann[1] and Marina Stolina[2*]

Table of contents

Supplemental Methods……………………………3

Supplemental Tables………………………………9

Supplemental Figures……………………………..11

Supplemental Methods

Tissue Collection

• Zenas Technologies (metadata in Supplemental Table 1) - The postmortem interval for all donors was reported to be within 3 hours. The specimens were removed from the approximate center mass of each chamber and represent transmural sections (e.g. epi to endo through the cardiac wall). All specimens were snap frozen to preserve RNA integrity. The normal (non-failing) donor population was defined as patients without clinical symptoms consistent with or previously diagnosed as heart failure. HFrEF donors were defined as patients with symptoms that categorized them as New York Heart Association (NYHA) functional Class II, III or IV, with LVEF < 50%, and a medical history consistent with heart failure.

• AnaBios Corporation (metadata in Supplemental Table 1) - AnaBios conducted a custom prospective collection in 2016. The warm ischemic time (WIT) was less than 1 hour, any reported downtime was less than 30 minutes, and the cold ischemic time (CIT) was less than 24 hours for all donors. Each region of the heart was submitted as three separate formats; snap frozen, OCT embedded, and formalin fixed paraffin embedded (FFPE). Normal (non-failing) donors excluded donors with any current or history of heart/cardiovascular disease (i.e. MI, CAD, stent(s), pacemaker, ventricular hypertrophy, arrhythmia, high cholesterol, plaques, heart defibrillation, murmur) and patients with inflammatory disease that may affect heart health such as systemic lupus erythematosus. The heart failure donors were defined to have had a history of heart failure (not necessarily cause of death) and excluded patients with inflammatory

diseases that affect heart health such as systemic lupus erythematosus (Supplemental Table 1).

• International Institute for the Advancement of Medicine (IIAM, metadata in Supplemental Table 1) - The warm ischemic time (WIT) was less than 1 hour, any reported downtime was less than 30 minutes, and the cold ischemic time (CIT) was less than 24 hours for all donors. Whole hearts were placed in one liter HTK or UW solution on wet ice. Hearts were dissected and each region of the heart was submitted as three separate formats; snap frozen, OCT embedded, and FFPE. The normal (non-failing) donors excluded donors with any current or past history of cardiovascular disease such as, high cholesterol, plaques, heart defibrillation, heart attack, pacemaker or heart murmur, myocardial infarction, coronary artery disease, stent(s), pacemaker, ventricular hypertrophy, arrhythmia and any inflammatory diseases that affect heart health such as systemic lupus erythematosus. The heart failure donors were defined to have had a history of heart failure (not necessarily cause of death) and excluded patients with inflammatory diseases that affect heart health such as systemic lupus erythematosus.

Frozen heart tissue pulverization and preparation for RNA extraction

Frozen heart tissue biopsies were pulverized using Bussmann Grinders, 100-200 mg of tissue powder was then homogenized for 30 seconds at 6500 rpm in buffer (350 µl of Qiagen RLT buffer with 1% BME; Qiagen, Germantown, MD, USA) using a MagNA Lyser (Roche Diagnostics, Indianapolis, Indiana, USA). After homogenization, 298 µl of RNase-free water and 2 µl of a 50 mg/ml proteinase K solution were added to each tube

and mixed well. Samples were incubated at 55°C for 10 minutes, centrifuged, and the supernatant was collected into a new 1.5ml RNase-free microcentrifuge tube.

RNA isolation and sequencing analysis of the heart

RNA extraction was performed using the RNeasy Micro Kit (Qiagen) with on-column DNase treatment (Qiagen) according to the manufacturer's instructions. RNA concentration and integrity were assessed using a NanoDrop 8000 (Thermo Fisher, Waltham, MA, USA) and a Bioanalyzer (Agilent, Santa Clara, CA, USA). Samples with ≥100 ng total RNA and RNA integrity numbers (RIN) ≥7 were used for sequencing. After passing the QC, total RNA (100 or 500 ng) was used for cDNA library preparation using a modified protocol based on the Illumina Truseq RNA sample preparation kit and the published method for strand-specific RNA-Seq[45,46]. After poly-A selection, fragmentation, and priming, reverse transcription was carried out for 1st strand cDNA synthesis in the presence of RNaseOut (Invitrogen) and actinomycin-D (MP Biomedicals). The synthesized cDNA was purified by using AMPure RNAClean beads (Beckman Coulter) following the manufacturer's protocol. A modified method by incorporation of dUTP instead of dTTP was prepared and used for the second strand synthesis according to previously published protocols[45,46]. After AMPure XP bead purification (Beckman Coulter), following the standard protocol recommended by the Illumina Truseq RNA kit, end repairing, A-tailing, and ligation of index adaptors were sequentially performed for generation of cDNA libraries. After size selection of libraries using Pippen Prep (SAGE Biosciences), the dUTP-containing strands were destroyed by digestion with USER enzymes (New England Biolabs) followed by a step of PCR enrichment for the introduction of strand specificity. After cleaning up, the enriched cDNA libraries were

analyzed in an Agilent Bioanalyser and quantified by Quant-iT^{TM} Pico-Green assays (Life Technologies) before being loaded onto the HiSeq platform (Illumina).

Heart tissue specificity

Heart tissue specificity was calculated using RNA-Seq of 30 different normal tissues in the GTEx database[22]. RNA-Seq datasets for normal tissues from GTEx were processed by Omicsoft based on human genome version GRCm38 and gene model GENCODE v24. Gene expression was represented as FPKM and normalized with a refinement of the commonly employed upper-quartile method[49] that sets the FPKM to a value of 10 at the 70th percentile[50,51]. In GTEx, different tissues include tissue subcategories, for example, uterus tissue include ectocervix, endocervix, and general uterus. To maintain gene expression profile in the tissue subcategories, median FPKM values were calculated for different tissue subcategories. Tissue gene expression used in the tissue specificity calculation was the highest median normalized FPKM value of tissue subcategories for that tissue. Heart enriched genes were selected as gene expression in heart as top 3 out of 30 normal tissues with normalized FPKM >=1.

Definition of subregion enriched gene lists

Based on the significance tests from DESeq2, genes enriched in a single chamber were significantly upregulated in that chamber in three pairwise comparisons with the other three heart chambers. For example, for LV enriched genes, they were significantly up-regulated in LV vs LA, LV vs RA and LV vs RV. Genes enriched in ventricles were significantly up-regulated in both ventricular chambers compared to both atria chambers in the pairwise comparisons. For example, up-regulated in LV vs LA, LV vs RA, RV vs LA and RV vs RA. Genes enriched in atria were significantly up-regulated in both atria

chambers compared to ventricular chambers in the pairwise comparisons. Genes enriched in left heart were significantly up-regulated in both left heart chambers compared to right heart chambers, for example, up-regulated in LV vs RA, LV vs RV, LA vs RA and LA vs RV. Genes enriched in right heart were significantly up-regulated in both right heart chambers compared to left heart chambers. These chambers-enriched categories may potentially overlap with each other. For example, in the results, we noticed some genes were both RA enriched, and atria enriched. For simplicity, if genes were both RA enriched and atria enriched, they were classified as atria enriched. No other overlapping chamber enriched categories were noticed.

Single-nucleus RNA-Seq (snRNA-Seq)

snRNA-Seq was performed on left ventricle samples from 4 NF and one HF donor using 10X Genomics Single cell Gene Expression 3' V3 chemistry (Catalog Number 1000075). Specifically, five 10 µm curls cut from OCT embedded human cardiac tissue were homogenized using a 2 ml Dounce homogenizer with 1 ml chilled lysis buffer (10mM Tris-HCl (pH 7.4), 10 mM NaCl, 3 mM MgCl2, 0.1% NP-40 and 0.2 U/ml RNase inhibitor). After filtering through a 40 µm cell trainer, nuclei were centrifuged at 700 x g for 5 min at 4°C. The nuclei were subsequently washed with 500 ml and then resuspended in 300 ml of Nuclei Wash/Resuspension Buffer (1x PBS with 2% BSA and 0.2 U/ml RNase inhibitor). 1 ml DRAQ5 (5 mM solution, Thermo Cat #62251) was added, and DRAQ5+ nuclei were sorted (70 mm nozzle on a BD Aria) into 50 ml Nuclei Wash Buffer (3 X 0.2 U/ml RNase inhibitor). Isolated nuclei were loaded into the 10x Genomics Chromium Controller for single nucleus microfluidic encapsulation and barcoding following manufacturer's instructions. Libraries were prepared per

manufacturer's instructions using 12 cycles for cDNA amplification and 13 cycles for indexing library amplification. Libraries were sequenced at 30x98 paired end on NovaSeq S4 flowcell. We used cell ranger (V3.1.0) mkref package to create a "pre-mRNA" reference with pre-built GRCh38 reference package, then we used cell ranger count pipeline to align the fastq reads and generated QC matrix and count matrix[61]. The Count matrix was further analyzed with Seurat (v3.2.0) R package[62] for batch effect correction by canonical correlation analysis, filtering (only nuclei with the number of detected genes between 200 and 4000, and the percent of mitochondrial reads less than 1% were kept for downstream analysis), normalization, variable features identification and dimensional reduction by Principal Component Analysis (PCA). The top 30 PCAs were used in graph-based clustering based on Louvain with resolution at 1.2, and cluster specific marker genes were identified with FindAllMarkers function by Wilcoxon Rank Sum test in Seurat, cell types were determined by cross-referencing with well-established cell type markers in literature[11,13]. Uniform Manifold Approximation and Project (UMAP) was used to visualize the high dimensional cell cluster distribution.

Supplemental Tables

Supplemental Table 1 Clinical characteristics of heart tissue donors

Group	Source	Sex M/F	Parameters (Mean ± SD)			
			Age (Y)	BMI Index	EF (%)	NYHA
Control (n=10)	Zenas Technologies	6/4	44.5 ± 9.9	33.1 ± 9.2	61.3 ± 8.0	N/A
HFrEF (n=12)		7/5	38.8 ± 13.0	25.9 ± 2.0*	34.9 ± 7.1****	2.8 ± 0.6
Control (n=3)§	AnaBios Corp	2/1	45.0 ± 12.0	22.3 ± 1.0	59.3 ± 5.7	N/A
Control (n=1)§	IIAM	1/0	18	28.4	N/A	N/A
HF (n=1)§		1/0	65	29.5	N/A	N/A

For Zenas Technologies samples: Control vs HFrEF: * $p < 0.05$; **** $p<0.0001$
Abbreviations: HFrEF, Heart Failure with reduced Ejection Fraction; M, Male; F, Female; Y, Year; SD, Standard Deviation; BMI, Body Mass Index; EF, Ejection Fraction; NYHA, New York Heart Association functional classification of heart failure. § Samples used for snRNA-Seq.

Supplemental Table 2. **Chamber-specific enrichment gene classification schema based on pairwise comparison of gene expression between heart chambers**

Chamber Specificity	**LA vs LV**	**LA vs RA**	**LA vs RV**	**LV vs RA**	**LV vs RV**	**RA vs RV**
LA Enriched	1	1	1	NE	NE	NE
LV Enriched	-1	NE	NE	1	1	NE
RA Enriched	NE	-1	NE	-1	NE	1
RV Enriched	NE	NE	-1	NE	-1	-1
Ventricles Enriched	-1	NE	-1	1	NE	-1
Atria Enriched	1	NE	1	-1	NE	1
Left Enriched	NE	1	1	1	1	NE
Right Enriched	NE	-1	-1	-1	-1	NE

1 as up-regulated, -1 as down-regulated, NE as not evaluated for the corresponding chamber enrichment test.
Abbreviations: LA, Left Atrium; RA, Right Atrium; LV, Left Ventricle; RV, Right Ventricle.

Supplemental Table 3. Heart- and subregion-enriched gene list (Excel Data Supplement S3) based on comparative analysis of RNA-Seq of 30 normal tissues in GTEx database and heart chambers of 10 non-failing hearts. The "Tissue Enriched" column presents the top 3 tissues (out of 30) where heart enriched genes

were highly expressed. The “Subregion Enriched” column presents the subregion where the gene is enriched (based on the pairwise comparison schema as shown in Supplemental Table 2).

Supplemental Table 4. Cell type enriched genes in NF heart tissue defined by snRNA-Seq (Excel Data Supplement S4)
The list is generated using the Seurat Package FindAllMarkers function aimed to compare major cardiac clusters. “pct.1”: The percentage of cells where the feature is detected in the specific cardiac cluster; “pct.2”: The average percentage of cells in all the other clusters where the feature is detected; “avg_logFC”: log fold-change of the average expression between the specific cluster and the rest of clusters. “p_val” and “p_val_adj”: raw and Bonferroni corrected p value using all features in the dataset based on Wilcoxon rank sum test.

Supplemental Table 5. Number of genes in each HFrEF regulatory group

Regulation	Gene Number
both down	615
both up	635
LA down LV up	6
LA only down	932
LA only up	1041
LA up LV down	10
LV only down	796
LV only up	943

Abbreviations: LA, Left Atrium; LV, Left Ventricle.

Supplemental Table 6. Significantly differentially expressed genes (DEGs) in HFrEF vs NF in LA heart tissue (Excel Data Supplement S6)
DEGs in LA were generated by DESeq2 Wald test to compare HFrEF vs NF two groups; “LA.HFrEF.log2FoldChange” indicates the log2 transformed fold change of gene expression comparing HFrEF vs NF in left atrium; “LA.HFrEF.padj” represents the BH (Benjamini-Hochberg) corrected p value based on the test.

Supplemental Table 7. Significantly differentially expressed genes (DEGs) in HFrEF vs NF in LV heart tissue (Excel Data Supplement S7)
DEGs in LV were generated by DESeq2 Wald test to compare HFrEF vs NF two groups; “LV.HFrEF.log2FoldChange” indicates the log2 transformed fold change of gene expression comparing HFrEF vs NF in left ventricle; “LV.HFrEF.padj” represents the BH (Benjamini-Hochberg) corrected p value based on the test.

Supplemental Figures

Supplemental Figure 1. Quality control summary of single nucleus RNA-Seq and chamber-enriched cell type markers expression. **(a)** bar graph presents the number of cells sequenced per sample; **(b)** box plot of the number of UMI counts per cell across the samples; **(c)** box plot of the number of detected genes per cell across the samples; **(d)** stacked bar chart showing the proportion of nuclei isolated from different cells across samples; **(e)** Heatmaps showing Z-score scaled expression of Heart- and Subregion-Enriched Cardiomyocytes, Cardiac Fibroblasts, Macrophages, Endothelium, Cardiac Neurons and Perivascular cells signature genes (Subregion-Enrichment fold change >2, BH adjusted $p < 0.05$) in each heart chamber in NF subjects by RNA-Seq.

Supplemental Figure 2. Proportion of cell types in bulk RNA-Seq cardiac tissue by deconvolution (top panel) and bulk RNA-Seq gene expression of a major cell type marker gene (bottom panel) across chambers **(a)** and disease groups **(b).**

Supplemental Figure 3. The two-dimensional principal subspace for all samples from non-failing and HFrEF diseased hearts based on genome-wide bulk RNA-Seq mRNA expression. Different chambers were in distinct colors and disease status are shown in square (HFrEF) and circle (Non-Failing). Blank represents sample from female donor and filled shape represents sample from male donor.

Supplemental Figure 4. Heatmaps showing expression of left heart (LA or LV) differentially expressed Cardiomyocytes, Macrophages, Cardiac Fibroblasts, Perivascular cells, Endothelium, Cardiac Neurons and Lymphocytes signature genes (fold change less than 0.5 or more than 2-fold, BH adjusted $p < 0.05$).

Supplemental Figure 5: Venn diagram comparing HFrEF altered genes in the LA and LV with Subregion-Enriched genes and Fulgent comprehensive cardiomyopathy clinical testing panel of 129 genes associated with hereditary cardiomyopathies including hypertrophic cardiomyopathy, dilated cardiomyopathy, left ventricular non compaction cardiomyopathy and hereditary arrhythmogenic right ventricular cardiomyopathy.

Supplemental Figure 1

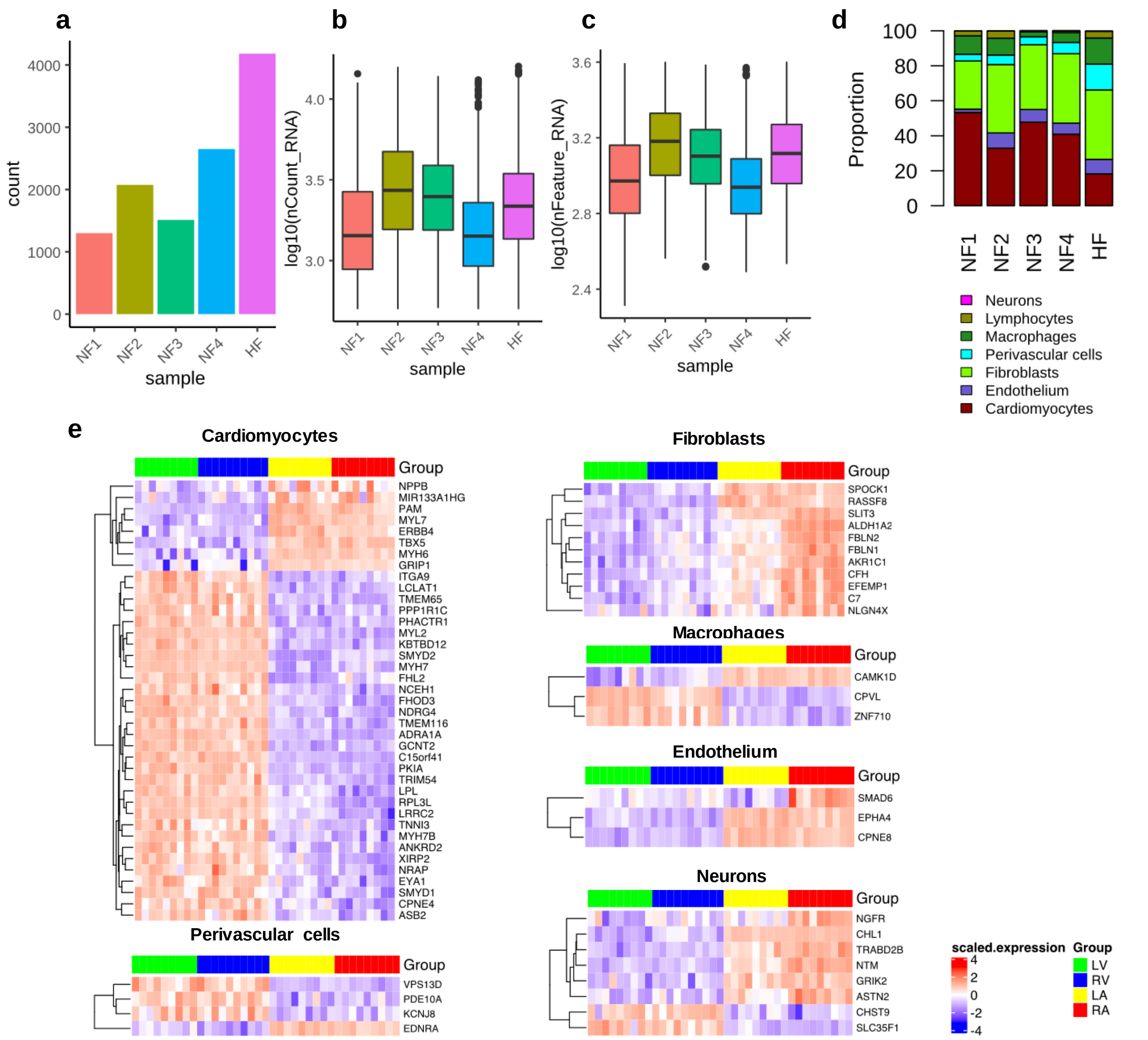

Supplemental Figure 2

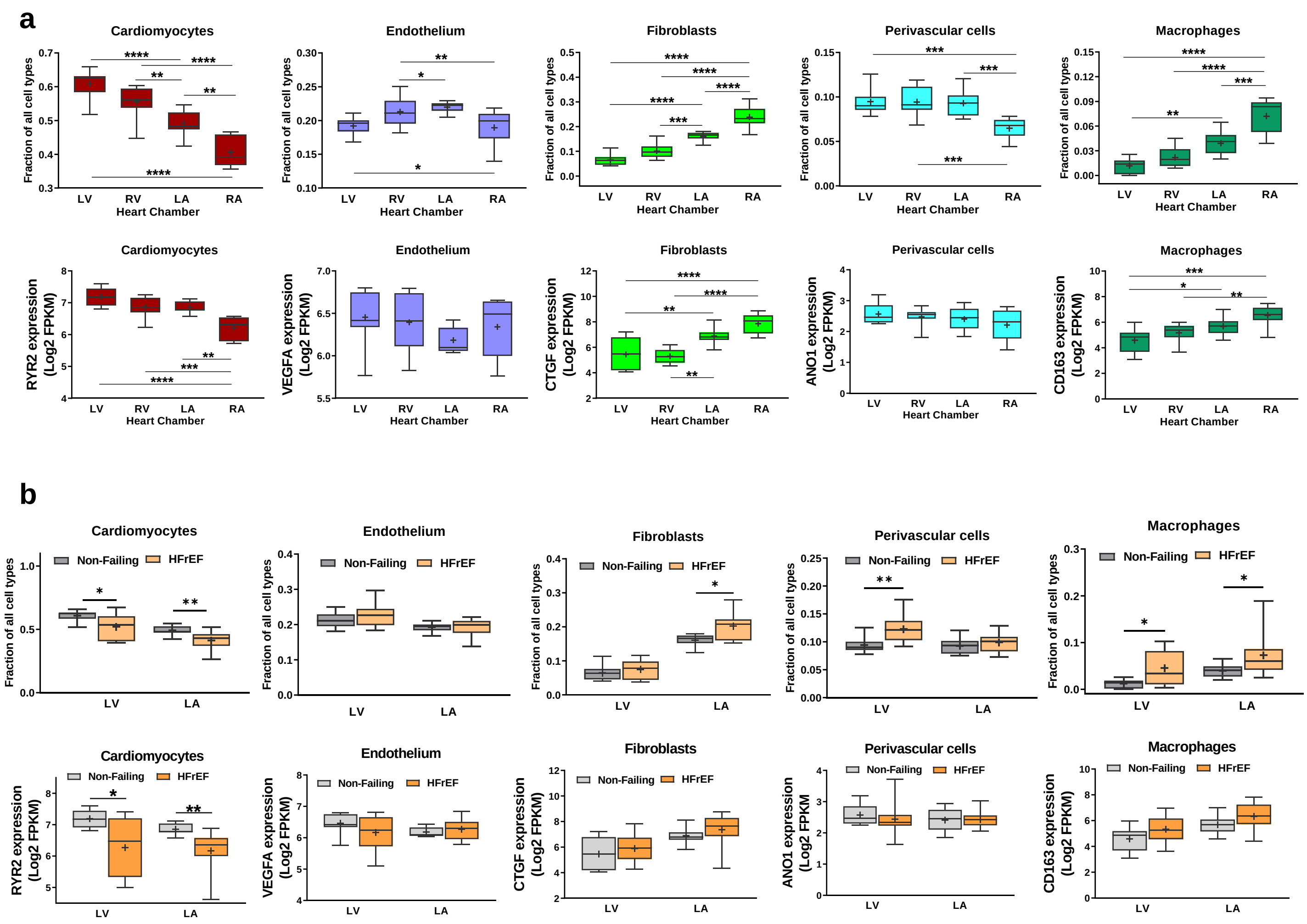

Supplemental Figure 3

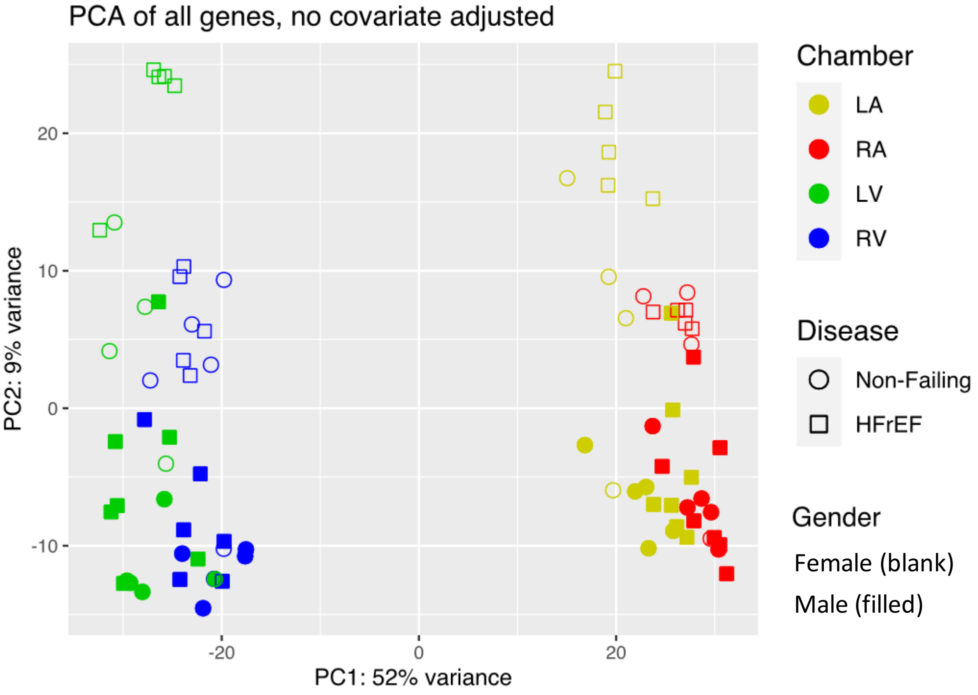

Supplemental Figure 4

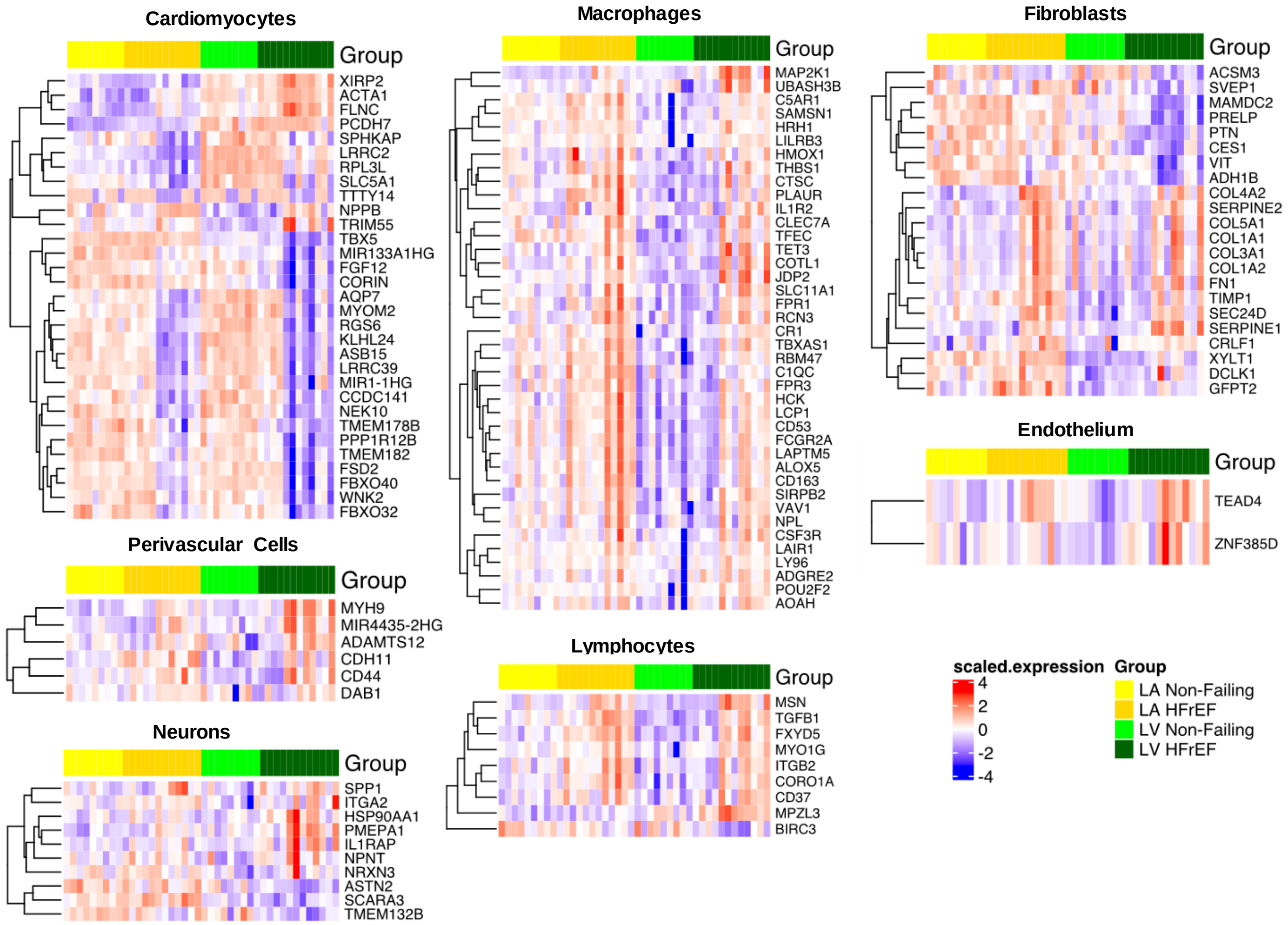

Supplemental Figure 5

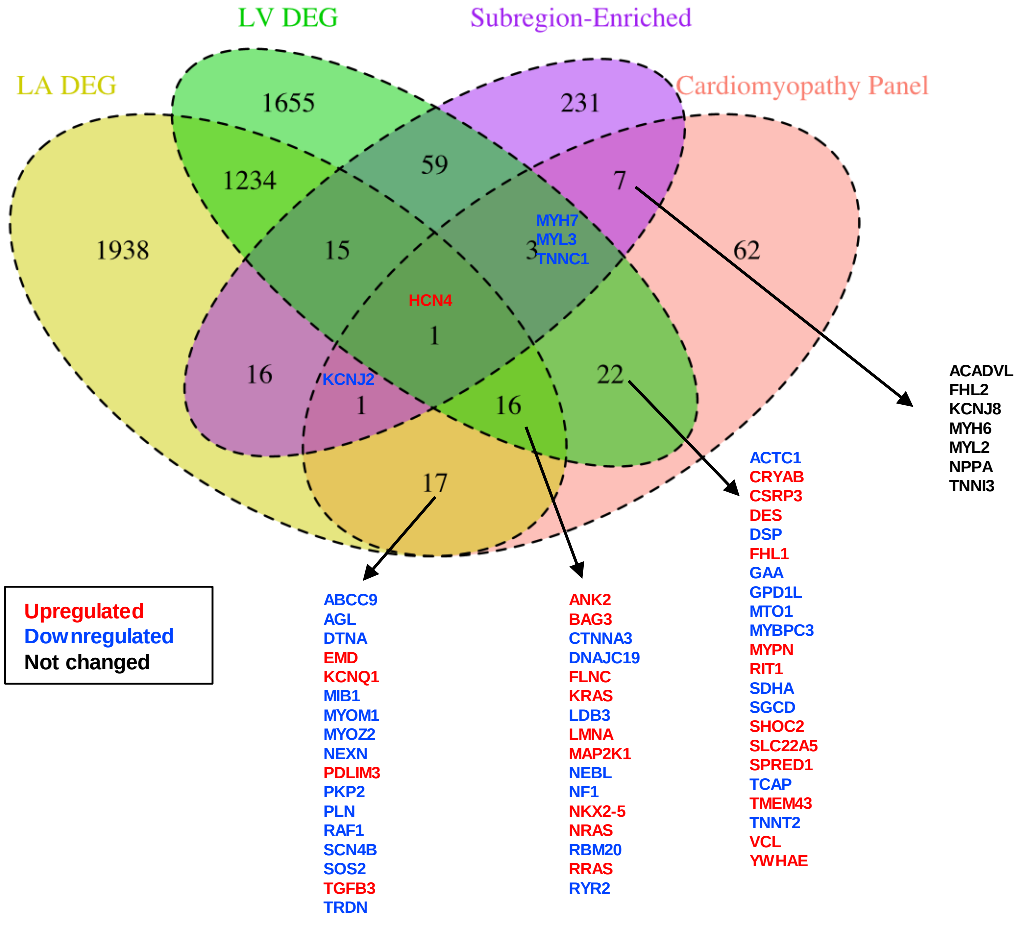

Supplement: Supplementary file 1 — Supplementary Information 1. [file 41598_2021_91214_MOESM1_ESM.pdf]
